# Cyclic Occurrence of Fire and Its Role in Carbon Dynamics along an Edaphic Moisture Gradient in Longleaf Pine Ecosystems

**DOI:** 10.1371/journal.pone.0054045

**Published:** 2013-01-15

**Authors:** Andrew Whelan, Robert Mitchell, Christina Staudhammer, Gregory Starr

**Affiliations:** 1 Department of Biological Sciences, University of Alabama, Tuscaloosa, Alabama, United States of America; 2 Jones Ecological Research Center, Newton, Georgia, United States of America; DOE Pacific Northwest National Laboratory, United States of America

## Abstract

Fire regulates the structure and function of savanna ecosystems, yet we lack understanding of how cyclic fire affects savanna carbon dynamics. Furthermore, it is largely unknown how predicted changes in climate may impact the interaction between fire and carbon cycling in these ecosystems. This study utilizes a novel combination of prescribed fire, eddy covariance (EC) and statistical techniques to investigate carbon dynamics in frequently burned longleaf pine savannas along a gradient of soil moisture availability (mesic, intermediate and xeric). This research approach allowed us to investigate the complex interactions between carbon exchange and cyclic fire along the ecological amplitude of longleaf pine. Over three years of EC measurement of net ecosystem exchange (NEE) show that the mesic site was a net carbon sink (NEE = −2.48 tonnes C ha^−1^), while intermediate and xeric sites were net carbon sources (NEE = 1.57 and 1.46 tonnes C ha^−1^, respectively), but when carbon losses due to fuel consumption were taken into account, all three sites were carbon sources (10.78, 7.95 and 9.69 tonnes C ha^−1^ at the mesic, intermediate and xeric sites, respectively). Nonetheless, rates of NEE returned to pre-fire levels 1–2 months following fire. Consumption of leaf area by prescribed fire was associated with reduction in NEE post-fire, and the system quickly recovered its carbon uptake capacity 30–60 days post fire. While losses due to fire affected carbon balances on short time scales (instantaneous to a few months), drought conditions over the final two years of the study were a more important driver of net carbon loss on yearly to multi-year time scales. However, longer-term observations over greater environmental variability and additional fire cycles would help to more precisely examine interactions between fire and climate and make future predictions about carbon dynamics in these systems.

## Introduction

Savannas are both ecologically and economically critical ecosystems at regional and global scales. Savannas cover nearly 1/3 of the earth’s land surface [1] and account for approximately 30% of the world’s primary productivity [2]. These ecosystems are important sources of food [2] and fiber [3], and thus, they are under increasing anthropogenic pressure that threatens their sustainability [1], [2], [4]. In addition to the economic benefits they provide, some savanna biomes, such as longleaf pine ecosystems are ecologically important as global “hot spots” for biodiversity [5], [6], [7]. Moreover, these ecosystems play an important role in the global carbon cycle, accounting for approximately 30% of the world’s terrestrial primary productivity [2]. Thus, it is ecologically and economically important to sustain savanna ecosystems [8].

Fire regulates the spatial and temporal controls of structure and function in savannas and open canopy woodlands. Using a dynamic global vegetation model, Bond and Keeley [9] reported that if fire was globally suppressed, the amount of closed canopy forest would more than double, increasing from 27% to 56% of the land surface, at the expense of savannas and open canopy woodlands. The loss of these ecosystems to closed canopy forests would result in diminished C_4_ grasses and biodiversity [9].

Fire also directly impacts the global carbon cycle by annually releasing an amount of CO_2_ to the atmosphere that is approximately half that emitted by combustion of fossil fuels [10]. Burning of savannas accounts for approximately 42% of the total carbon released annually from anthropogenic and naturally occurring fire [2], [4]. The impact that fire has on carbon cycling in these ecosystems, however, is much more complex than the initial release of CO_2_. Fire also alters vegetation and affects plant resources such as light, water and nutrients [11]. Fire interacts with vegetation and the environment directly and indirectly to influence carbon cycles. Indirectly, fire increases heterotrophic and autotrophic respiration [12]. In open canopy ecosystems many plants survive fire but must replace lost leaf area or resprout entirely; thus autotrophic respiration increases to rebuild biomass. Furthermore, plant biomass that was killed but not directly consumed by fire falls to the soil surface and fuels heterotrophic respiration. Higher soil temperatures associated with decreased post-fire albedo [13] and higher soil pH due to cation release [14] can also accelerate rates of decomposition and carbon efflux.

Carbon assimilation can also be strongly affected by fire [15]. Even low intensity ground fires can scorch tree crowns, and can often completely consume shrub or small tree crowns. Recovery of functional leaf area occurs quickly through sprouting and crown reconstruction [16]. Concomitantly, net ecosystem uptake of CO_2_ can recover shortly following fire [12]. However, we have little information on how climate affects rates of recovery, and how those rates vary among fires. Further, fire interacts in complex ways with climate to regulate the temporal and spatial variation in carbon pools and fluxes [17]. Changes in precipitation can affect productivity and fuel accumulation, which can affect fire intensity, the amount of carbon released by fire, and post-fire recovery [17].

While we understand that fires occur cyclically and return at frequencies based on fire regimes [18], [19], the impact that fire has on carbon dynamics has often been viewed as a single event [20]. The lack of research on carbon dynamics over multiple fire cycles is particularly salient in frequently burned savanna ecosystems. Because these ecosystems are highly productive and are able to store greater amounts of carbon when fire is suppressed, some have recommended that increasing the fire return interval may be an effective strategy for global carbon sequestration [2], [12]. However, the increased fuel loads associated with lengthened fire return intervals increase the risk of high intensity wildfire, which may result in greater emissions of carbon to the atmosphere [21]. Furthermore, increased fire intensity associated with wildfire increases the potential for overstory mortality, which results in additional loss of carbon from the standing crop and a reduction in ecosystem capacity to assimilate carbon for years [22], [23]. To better formulate strategies of carbon management in savannas it is necessary to develop a much more robust understanding of the controls on and drivers of temporal and spatial variation of the carbon cycle.

Longleaf pine savannas are good models to investigate how carbon cycles are regulated in frequently burned, open canopy ecosystems. They feature the archetypal savanna structure of a C_3_ overstory and a C_4_ dominated understory [24]. Longleaf pine savannas also occur along a wide edaphic gradient based on soil moisture availability, from xeric sandhills to more mesic flatwoods [25]. Low intensity fires that burn every 1 to 3 years maintain a biodiverse contingent of highly productive, fire adapted vegetation [26]. However, little is known about how multiple fire cycles impact spatial and temporal controls on carbon cycling at the ecosystem scale.

In this study, we used a combination of eddy covariance measurements and collection of pre- and post-fire vegetation and litter samples on three sites along a soil moisture gradient to answer the following questions regarding the interaction of soil water availability and fire:

a) How does fire influence net ecosystem exchange (NEE), gross ecosystem exchange (GEE), and ecosystem respiration (R_eco_), and how long do these variables take to recover post-fire? b) Does soil water holding capacity influence how fire affects NEE, GEE, and R_eco_?
b) How much carbon is released by prescribed fire and does this amount vary with soil water holding capacity? b) How do differences in soil water holding capacity affect water use efficiency in longleaf pine ecosystems?

## Materials and Methods

### Study Site

This study was conducted from October 22, 2008 to October 22, 2011 at three sites located at the Joseph W. Jones Ecological Research Center (JJERC) in southwestern Georgia, USA (31.2201°N, 84.4792°W). The Robert W. Woodruff Foundation privately owns the lands and Dr. Lindsay Boring, director of JJERC, gave permission for use of these sites. This area is part of the southeastern coastal plain, and is characterized by irregular karst topography [25]. The climate is humid subtropical with mean annual precipitation of 1310 mm spread evenly throughout the year [27]. Mean monthly minimum and maximum temperature extremes range from 3° to 16°C in the winter and from 22° to 33°C in the summer [28].

Based on differences in soil drainage classes, three study sites were selected that encompass the range of soil moisture availability at the JJERC. The mesic site lies on somewhat poorly drained sandy loam over sandy clay loam or clay textured soils. Soils are classified as Arenic Paleudults and have an argillic horizon within 95 cm of the soil surface [25]. The intermediate site is ∼9.5 km southeast of the mesic site, and lies on a well-drained upland terrace with depth to the argillic horizon of approximately 165 cm. Soils are loamy sand over sand loams and are classified as Typic Hapludults or Typic, Arenic and Grossarenic Paleudults [25]. The xeric site, located 8 km north of the intermediate site, is excessively well drained and lies on deep sandy soils with no argillic horizon (no clay accumulation in the upper 300 cm of soil). Soils at the xeric site are classified as Typic Quartzipsammants [25]. These differences in soil water holding capacity affect the overlying vegetation structure and species composition.

The mesic, intermediate and xeric sites are all dominated by longleaf pine (*Pinus palustris* Mill.) in the overstory and the perennial C_4_ grass species, wiregrass (*Aristida stricta* Michx.), in the understory; however, the composition and abundance of other overstory and understory species varies with soil moisture holding capacity [24]. The mesic site is dominated by longleaf pine in both the overstory and midstory. *Diospyros virginiana* L. is common and occurs as a shrubby component of the understory [29]. These sites have previously been described by Mitchell et al [26], Kirkman et al [24] and Ford et al [30]. The overstory at the intermediate site is dominated by longleaf pine with little intrusion by other species. *Quercus* (*Q*.) *incana* Bartr. and *Q. margaretta* (Ashe) Small occur only in the midstory and understory. At the xeric site, the overstory is dominated by longleaf pine but a large component of the scrub oak species *Q. laevis* Walt. and *Q. margaretta* occur in the overstory, midstory and understory.

### Fire Regime

Longleaf pine ecosystems have one of the highest rates of fire frequency in the USA [31] and the world [32], and high biodiversity contributes to their unique structure and function [33]. Longleaf ecosystems cannot exist without fire and when fire is suppressed for as little as 4 years the ecosystem loses biodiversity, structure and function [34]. For this reason our study does not include a “control site” where fire is excluded.

During this study, each site was burned in January 2009 and again in March 2011 (Table 1). Prior to this study, the mesic and xeric sites had last been burned in the winter of 2007. The intermediate site had been on a different burn schedule and was last burned in the winter of 2008. Prescribed fires were conducted as follows: The downwind side of the unit was ignited, and fire was allowed to back into the unit creating an additional buffer between the firebreak and the rest of the unit. Depending on fuel loads and local weather conditions, strip head fires perpendicular to the wind were then ignited every 30–50 meters upwind of the backing fire. This allowed the fires to consume the fuels in the units, but kept fire intensity low, and minimized damage to overstory trees.

**Table 1:** Precipitation and carbon fluxes over the study period.

Site	Year	Precipitation	NEE	Reco	GEE	Reco/GEE(%)
Mesic	1	1474	−.35	18.30	19.65	93.15
	2	1181	−1.06	16.58	17.64	93.97
	3	766	−0.07	13.52	13.60	99.46
	Total	3421	−2.48	48.40	50.88	95.12
Intermediate	1	1275	0.53	18.69	18.15	102.94
	2	835	0.69	16.54	15.85	104.37
	3	741	0.35	15.10	14.76	102.36
	Total	2851	1.57	50.33	48.76	103.23
Xeric	1	1361	1.24	17.19	15.96	107.75
	2	1018	0.15	15.44	15.29	100.99
	3	755	0.07	13.74	13.67	100.55
	Total	3134	1.46	46.38	44.92	103.26

Annual estimates and totals over the study period for precipitation (mm), NEE, R_eco_, and GEE (tonnes C m^−2^) for the mesic, intermediate, and xeric sites.

### Flux Measurements

NEE was measured continuously at all three sites from October 2008 to October 2011 using open-path eddy covariance techniques [35], [36]. By applying a control volume approach, NEE was estimated through a simplification of the continuity equation (Eq. 1). The vertical rate of change of mean molar CO_2_ concentration and the vertical scalar flux divergence from ground level to the measurement height (*z*, *m*) are represented by integrals I and II in Eq. 1, respectively [37].
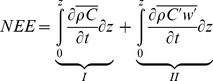
(1)where *ρ* is the density of dry air, *C* is CO_2_ concentration (µmol CO_2_ m^−3^) and *w* is the vertical wind velocity (m s^−1^). Primes denote instantaneous fluctuation (at 10 hz) about the mean, and overbars denote the mean over the averaging period, which was 30 min in this case. CO_2_ is stored directly beneath the eddy covariance instrumentation and was calculated as a function of mean molar CO_2_ concentration and measurement height. CO_2_ concentration and the vertical velocities are measured at a fixed plane above mean canopy height. In this study, micrometeorological convention was used, where negative fluxes represent ecosystem uptake of carbon. CO_2_ and water vapor concentration were measured with an open path infrared gas analyzer (IRGA, LI-7500, LI-COR Inc., Lincoln, NE), and three dimensional windspeed and air temperature were measured with a three dimensional sonic anemometer (CSAT3, Campbell Scientific, Logan, UT). These sensors were installed approximately 4 m above mean canopy height at each site (34.5, 37.5, and 34.9 m for the mesic, intermediate and xeric sites, respectively). The sonic anemometer and the IRGA were placed approximately 0.2 m apart in order to minimize flow distortion between the two instruments. The optical path of the IRGA was vertically aligned to match the sampling volume of the sonic anemometer. Data were logged on CR-3000 dataloggers (Campbell Scientific, Logan, UT) and stored on 1 GB CompactFlash cards. The IRGA was calibrated monthly using dry N_2_ gas, a gas mixture with a known concentration of CO_2_, and a dew point generator (LI-610, LI-COR Inc., Lincoln, NE).

### Fuel Consumption

To determine the amount of carbon lost from the ecosystem during the fires, we sampled above ground litter and biomass before and after the fires using the methods described by Ottmar [38]. The number of clip plots sampled varied from 10 to 20, such that the standard error of the mean was <15% of the mean value. Prior to burning in 2009, all above ground litter, herbaceous fuels and woody plants <1 m in height were collected from 0.75 m^2^ plots located within the footprints of the eddy covariance towers at the mesic and xeric sites. The procedure was the same at the intermediate site, except biomass was harvested from 1 m^2^ clip plots. Within one month following prescribed burning in 2009, above ground biomass was harvested from 4 m^2^ clip plots at the mesic and xeric sites. Pre- and post fire clip plots were not paired at the mesic and xeric sites in 2009, because post-fire biomass was uniformly very low. Therefore, we used these unpaired pre- and post-fire clip plots as a descriptive measure of carbon lost to fire at these two sites without statistical tests. At the intermediate site, post-fire above ground biomass was harvested from 1 m^2^ clip plots located directly adjacent to the pre-fire clip plots, which yielded a paired sampling design. In 2011, the sampling design was more uniform between the sites. Above ground biomass was harvested pre- and post-fire from paired 1 m^2^ clip plots at each site. Clip plots at all sites were located every 25 m along transects that started at the base of each tower and extended within the flux footprint either windward or leeward in the direction of the prevailing wind. Harvested clip plot litter and biomass was dried to a constant weight and mass. Carbon content was assumed to be 50% of the dry weight of the litter and biomass. Fuel consumption was the difference of pre-burn and post-burn dry weight.

### Water Use Efficiency

To answer questions about how soil water holding capacity and fire affect water use efficiency (WUE), we investigated the relationship between evapotranspiration (ET; mm H_2_O s^−1^) and carbon fixed through photosynthesis (GEE; g C m^−2^ s^−1^) before and after the fires. We used the following formula to calculate WUE [39]:
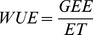
(2)


ET was calculated for each half hour period using the following formula:
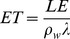
(3)where *LE* is latent energy measured by eddy covariance (W m^−2^), *ρ_w_* is the density of water (Kg m^−3^), and *λ* is the latent heat of vaporization of water (KJ Kg^−1^). This allowed us to examine the relationship between GEE and ET pre- and post-fire, and to determine whether changes in WUE were more affected by GEE or ET.

### Meteorological Instrumentation

In addition to flux data, meteorological data were also collected and stored on the CR3000 datalogger (Campbell Scientific, Logan, UT). Meteorological data measured on the towers included: photosynthetically active radiation (PAR, LI-190, LI-COR Inc., Lincoln, NE), global radiation (Rs LI-200SZ, LI-COR Inc., Lincoln, NE), four component net radiation (Rn, NR01, Hukseflux, thermal sensors, Delft, The Netherlands), precipitation (TE525 Tipping Bucket Rain Gauge, Texas Electronics, Dallas, TX), wind direction and velocity (Model 05103-5, R.M. Young, Traverse City, MI), air temperature (T_air_) and relative humidity (HMP45C, Campbell Scientific, Logan, UT), and barometric pressure (PTB110, Vaisala, Helsinki, Finland).

Soil temperature, volumetric water content of the soil and soil heat flux were measured in one location near the base of the tower at each site every 15 s and averaged every 30 min on an independently powered CR10X datalogger. Soil temperatures were measured at depths of 4 and 8 cm with insulated thermocouples (Type-T, Omega Engineering, INC., Stamford, CT). Soil heat flux was measured at a depth of 8 cm with soil heat flux plates (HFP01, Hukesflux, Delft, The Netherlands). Volumetric water content was measured within the top 20 cm of the soil surface using a water content reflectrometer probe (CS616, Campbell Scientific, Logan, UT).

### Data Processing

Raw EC data were processed using EdiRe (v.1.4.3.1184; [40]), which carried out a 2-d coordinate rotation of the horizontal wind velocities to obtain turbulence statistics perpendicular to the local streamline. The covariance between turbulence and scalar concentrations was maximized through examination of the time series at 0.1 s intervals on both sides of a fixed lagtime (in this case, ∼ 0.3 s). Because of the relatively short roughness lengths and uniform canopy structure at these sites, we assumed that the influence of coherent structures and low frequency effects were captured by this approach. Fluxes were calculated for half-hour intervals and then corrected for the mass transfer resulting from changes in density not accounted for by the IRGA [41], [42]. Barometric pressure data were used to correct fluxes to standard atmospheric pressure.

Flux data screening was applied to eliminate 30-min fluxes resulting from systematic errors such as: *i*) rain and condensation in the sampling path, *ii*) incomplete 30-min datasets during system calibration or maintenance, *iii*) poor coupling of the canopy with the external atmospheric conditions, as defined by the friction velocity, u*, using a threshold <0.20 m s^−1^ [43], [44], and *iv*) excessive variation from the half-hourly mean based on an analysis of standard deviations for *u, v*, and *w* wind and CO_2_ statistics. Quality assurance of the flux data was also maintained by examining plausibility tests (*i.e*., NEE<−30 and NEE >30 µmol m^−2^ s^−1^), stationarity criteria, and integral turbulent statistics [45], [46].

Eddy covariance measurements of CO_2_ estimate net ecosystem exchange at a time resolution of one hour or less [37], [47], such that:
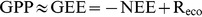
(4)where: GPP is gross primary production. GPP cannot be measured directly, but rather is estimated from the right hand terms in Eq. 4. Half hourly fluxes of NEE (µmol m^−2^ s^−1^) were used to calculate GEE and R_eco_ in g C m^−2 ^s^−1^ from Eq. 4 following Randerson et al. [48], Loescher et al. [37] and Campbell et al. [49].

Missing half hourly data were gap-filled using separate functions for day and night (*NEE_day_, NEE_night_*). When photosynthetically active radiation (PAR) was ≥10 µmol m^−2^ s^−1^_,_ daytime NEE data were gap-filled using a Michaelis-Menten approach,
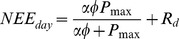
(5)and, when PAR was <10 µmol m^−2^ s^−1^, nighttime NEE data were gap-filled using a modification of Lloyd and Taylor 1994 approach,
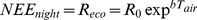
(6)where: α is the apparent quantum efficiency (-µmol CO2 µmol quanta−1), φ is PAR (µmol quanta), Rd is ecosystem respiration (µmol CO2 m−2 s−1), Pmax is the maximum ecosystem CO2 uptake rate (µmol CO2 m−2 s−1), R0 is the base respiration rate when air temperature is 0°C, and b is an empirical coefficient. These functional relationships were calculated on a monthly basis to gap-fill the data where enough data were available. Where too few observations were available to produce stable and biologically reasonable parameter estimates, annual equations were used to gap-fill data by site (3, 4, and 5 months at the mesic, intermediate and xeric sites, respectively, for NEEday, and 11 months at the mesic and xeric sites, and 14 month at the intermediate site for NEEnight).

Gap-filled data accounted for 33%, 28% and 32% of daytime, and 63%, 52% and 66% of nighttime values for mesic, intermediate and xeric sites, respectively. The percentage of gap filled data was within the range found in EC studies for daytime data [50], but slightly higher than others for nighttime data due to atmospheric stability conditions at the sites; however, it has been shown that daily and annual calculations of CO_2_ fluxes are very robust under this methodology [48].

Error estimations from gap-filled values of NEE were performed via bootstrap methods [51]. Although Monte Carlo methods have been widely used in this context [52], [53], synthetic data generation and parameter distribution determination via bootstrap methods are more efficient when the distribution of the measurement error is unknown [54], [55]. For an original dataset of size *n*, synthetic datasets are generated by randomly selecting *n* observations with replacement from the original data. We generated 1000 synthetic datasets for each estimated gap-filling model (day and night models in Eqs. 5 and 6, respectively, on a monthly or annual basis where appropriate), and constructed the distribution of each model parameter. These distributions were then checked to ensure that the model parameters derived from the original data were contained within a 90% confidence region. Because 90% confidence regions cover a smaller range, they are more stringent than 95% confidence regions, and offer greater assurance that model parameters were unbiased. In all cases, parameter estimates from the original data were within the 90% bootstrap confidence regions (Table S.1 and S.2, Supplementary Information).

### Statistical Analysis

To mitigate for multicollinearity and to select an initial set of predictor variables for modeling, correlations among all environmental and carbon flux variables in the raw, half-hourly data were investigated by site by computing simple Pearson correlation coefficients. These correlations were analyzed descriptively because high autocorrelation in the data renders significance tests meaningless [56]. PAR and net radiation were highly correlated with each other, and had strong correlations with NEE, GEE and soil heat flux. T_air_ had strong correlations with soil temperature at both 4 and 8 cm, soil heat flux, GEE and R_eco_. Accordingly, net radiation, soil heat flux, and soil temperatures at both 4 and 8 cm were excluded from all subsequent analysis.

To answer question 1a, 1b, and 2a, general linear models were formulated to identify which environmental variables were significant drivers of carbon exchange at each site, and to show changes in NEE, GEE and R_eco_ over the fire cycle. Because half-hourly data have strong temporal autocorrelation over long time spans, half-hourly data were averaged over different time periods and tested for autocorrelation via the Durbin-Watson test. A data averaging period of 28 days mitigated significant temporal autocorrelation. Accordingly, all subsequent statistical analyses were performed on data averaged over 28 days. Initial general linear models for NEE and GEE included the following environmental variables: PAR, T_air_, volumetric water content of the soil (VWC), vapor pressure deficit (VPD) and windspeed (WS). Because respiration is not strongly affected by light, PAR was excluded from the model for R_eco_. In addition to environmental variables, categorical variables for site and fire cycle time (FCT) were also included. FCT 1 included data in the first 28 days following fire, FCT 2 and 3 corresponded to the next two 28 day periods, FCT 4 represented the next 140 days, FCT 5 the next 224 days, and “pre-fire” the approximately 336 days before the next fire. Interactive effects between categorical and selected environmental variables were also included in the initial models based on observations in previous studies and personal observations [26], [57]. The interactive effects included in initial models were: FCT x PAR, T_air_, soil moisture, VPD and WS, and site x PAR, soil moisture and WS. We then successively eliminated the least significant effects through backwards elimination, dropping effects until only significant effects remained. We also verified at each elimination step that removing the variable resulted in a better (lower) value of the Akaike Information Criteria. Where an interaction was significant, the underlying simple effects were kept in the model, regardless of significance. Least square means estimates of significant interactive effects were generated and differences tested via Scheffe’s test to determine the magnitude, direction, and significance of effect levels where appropriate.

We further investigated how soil water holding capacity and fire interact to drive carbon dynamics by modeling the responses of NEE to light and temperature at each site. Daytime light response curves and nighttime temperature response curves were fit to the non-missing (i.e., non-gap-filled) data over 28 day pre- and post-fire periods using gap-filling equations (Eq. 5 and 6, respectively). Differences in NEE by site, and pre- and post-fire were modeled via indicator variables, which directly represented parameter differences by site, and pre- and post-fire. Parameters estimated were apparent quantum yield (*α*), the maximum carbon uptake rate (*P_max_*), and the base respiration rate at zero PAR (*R_d_*). *α* and *P_max_* affect the shape of the light response curve, with lower values of either resulting in a flatter curve that is nearer to its y-intercept. *R_d_* represents the curve’s y-intercept, the base respiration rate when PAR is 0. Additionally, these parameter estimates were used to generate representative light and temperature response curves that helped to visually describe how fire affected NEE and R_eco_.

We calculated fuel consumption in each fire for all three sites, and summed the results with NEE over the study period to obtain values for net biome exchange (NBE). For each fire, analysis of variance tests were used to determine if there were significant differences in fuel consumption among sites. Fuel consumption in each fire was also compared within site with the explicit realization that results for the 2009 fires could not be tested statistically.

We investigated WUE (question 2b) by formulating GLMs with volumetric water content of the soil (VWC), ET and GEE as predictor variables. Initially, these three variables and their interactions with site were included in the model as simple and interactive effects. Least significant effects were eliminated from the model successively until only significant effects remained. To elucidate how ET and WUE efficiency are affected by stomatal dynamics, we also modeled the effects of VPD on ET. All analyses were performed using SAS (Version 9.2) with a type I error level of 0.05.

## Results

### Environmental Conditions

**Figure 1:**
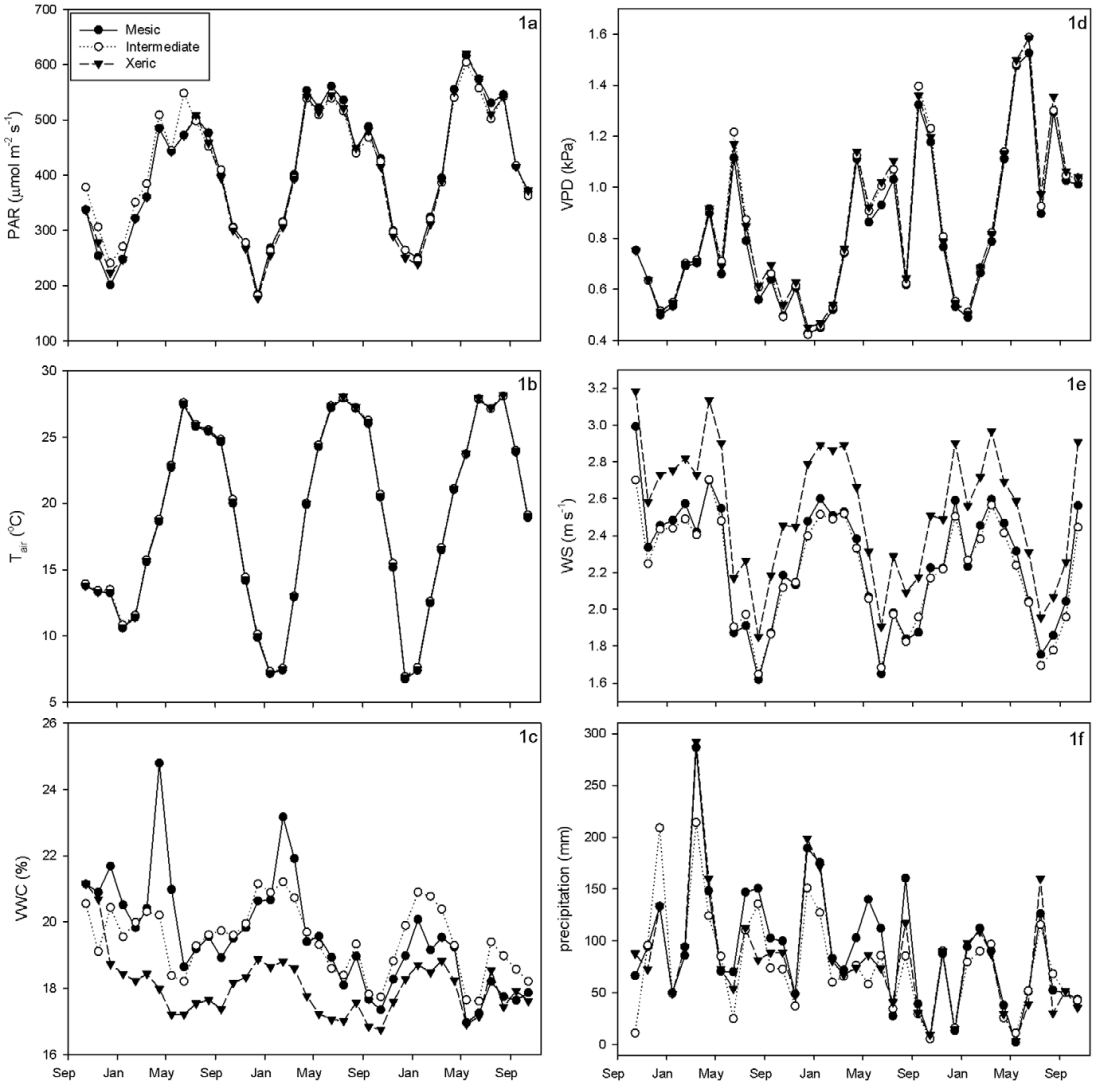
Environmental variables over the study period. Environmental variables measured at xeric (closed triangles, dashed lines), intermediate (open circles, dotted lines) and mesic (closed circles, solid lines) sites at the Jones Center from October 2008 to October 2011. Monthly means were calculated for: (a) photosynthetically active radiation (PAR), (b) air temperature (T_air_), (c) volumetric water content of the soil (VWC), (d) vapor pressure deficit (VPD), and (e) windspeed. Monthly sums were calculated for (f) precipitation.

Volumetric water content (VWC) of the soils varied among sites as targeted by the study design; however, rainfall, temperature, and relative humidity were similar among sites as they were separated by less than 10 km (Figure 1). Annual precipitation amounts during the first year of the study were near the long-term annual mean while the two following years were well below average [28]. (Table 1, Figure 1f). Mean monthly and annual air temperatures (T_air_) corresponded well to long-term temperature patterns; the minimum mean monthly temperature of 6.7°C at the mesic site during December 2010 was close to the long-term monthly minima for the area of 9.1°C and the maximum mean monthly temperature of 28.0°C at the xeric site in July 2010 (Figure 1b). was similar to the long-term mean monthly maxima of 27.2°C [28]. Mean T_air_ over the three year study period was 18.9°C, which correspond well with the long-term mean annual temperature of 18.7°C [28]. PAR followed a seasonal pattern with slight differences attributed to local cloud cover (Figure 1a). Vapor pressure deficit (VPD) also followed a seasonal pattern with the lowest values occurring during the winter months and higher values occurring during the summer at all sites (Figure 1d). Windspeed followed a seasonal pattern that was generally similar at the mesic and intermediate sites, and consistently higher at the xeric site (Figure 1e).

### Carbon Balance

**Figure 2:**
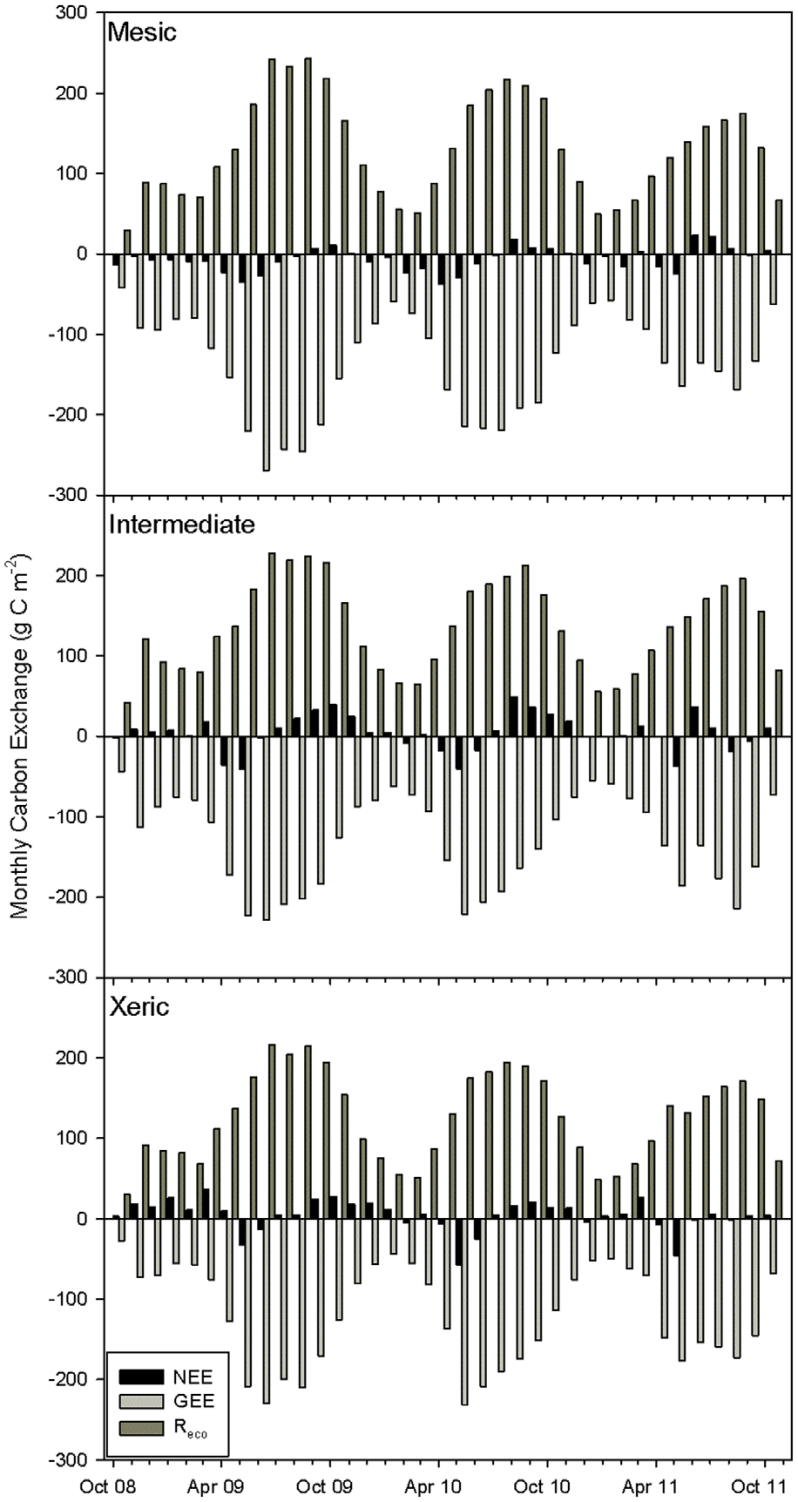
Monthly NEE, GEE and R_eco_ over the study period.

Over the study period, the mesic site was a small carbon sink (NEE = −2.48 tonnes C ha^−1^), while the intermediate (previously burned in 2008) and xeric sites were both sources of carbon (1.57 and 1.46 tonnes C ha^−1^, respectively; Table 1, Figure 2). When carbon lost during fire was incorporated into the carbon budget, estimates of net biome exchange (NBE) showed all sites to be large carbon sources (Table 2). NBE over the period between the 2009 and 2011 burns tells a similar story. The mesic site was a net carbon sink, and the intermediate site was a larger source than the xeric site. Over this interval, the mesic and xeric sites were sources of similar strength (0.04 and 0.05 tonnes C ha^−1^, respectively), while the intermediate site was a slightly larger source (0.07 tonnes C ha^−1^).

**Table 2:** NEE, fuel consumption and net biome exchange (NBE) (tonnes ha^−1^) over the study period.

	Mesic	Intermediate	Xeric
Total NEE	−2.483	1.575	1.462
2009 fuel consumption	6.807	0.506	4.838
2011 fuel consumption	6.455	5.869	3.390
Net Biome Exchange	10.780	7.950	9.690

Total NEE values are the sums of annual NEE from the three years of the study. NBE is the sum of total NEE and fuel consumption during the two fires.

Fuel consumption during both the 2009 and 2011 fires was greatest at the mesic site (Table 2). The intermediate site lost less carbon during the 2009 fire than the xeric site (Table 2). Fuel consumed during the 2011 fire was significantly lower at the xeric site than at the mesic site (p = 0.0126). Fuel consumed during the 2011 fire was lower at the xeric site than at the intermediate site; however, this difference was not significant (p = 0.0521, Table 2).

### Environmental Drivers of Carbon Fluxes

**Figure 3:**
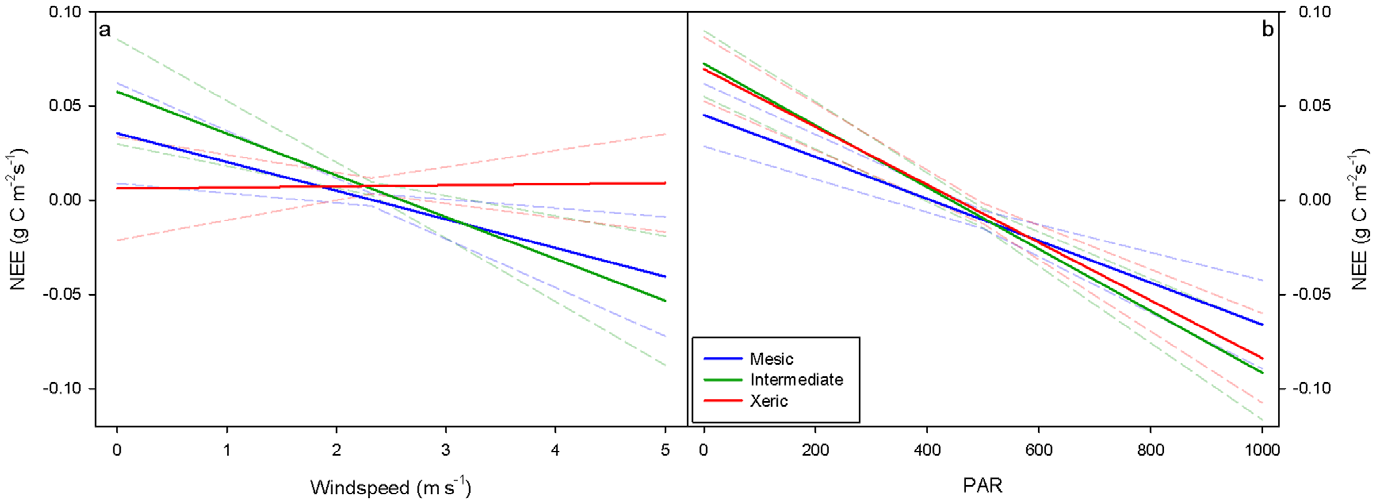
Interactive effect of Windspeed*Site and PAR*Site on NEE. Least square mean values of NEE at the xeric, intermediate and mesic sites over (a) a range of wind speeds, and (b) a range of PAR values. FCT 1 is the first 28 days following fire, FCT 2 and 3 are the next two 28 day periods, FCT 4 represents the next 140 days, and FCT 5 represents the next 224 days. To mitigate for autocorrelation PAR and NEE are 28 day means.

A general linear model identified the simple effects T_air_, and VPD, as well as the interactive effects of site x PAR, FCT x PAR, and site x WS to be significant variables in explaining variation in NEE (Table 3). Increases in PAR resulted in higher C uptake (carbon fixed through photosynthesis) and lower NEE values, although the magnitude of that effect varied with FCT (p = 0.0001) and site (p = 0.037). Increases in T_air_ and VPD had the opposite effect, reducing carbon uptake (p = 0.0001 and p* = *0.0004, respectively). The effect of WS was to increase carbon uptake at the mesic and intermediate sites, while it had the opposite effect at the xeric site (Figure 3a; p* = *0.0188).

**Table 3:** Type III tests of fixed effects for the models of NEE, R_eco_ and GEE.

Effect	Num. DF	Den. DF	F Value	Pr>F
*Model of NEE*				
Site	2	96	5.06	0.0082
FCT	5	96	9.11	<.0001
PAR	1	96	46.30	<.0001
T_air_	1	96	16.21	0.0001
VPD	1	96	13.61	0.0004
WS	1	96	5.94	0.0166
PAR*Site	2	96	3.41	0.037
WS*Site	2	96	4.14	0.0188
PAR*FCT	5	96	5.69	0.0001
*Model of R_eco_*				
Site	2	112	4.54	0.0127
T_air_	1	112	1048.03	<.0001
VPD	1	112	91.50	<.0001
*Model of GEE*				
FCT	5	102	4.42	0.0011
PAR	1	102	6.62	0.0115
T_air_	1	102	82.03	<.0001
VWC	1	102	11.98	0.0008
VPD	1	102	12.29	0.0007
VPD*FCT	5	102	4.16	0.0018

Tables include for each effect, the degrees of freedom in the numerator (Num. DF), degrees of freedom in the denominator (Den. DF), and the value of the F statistic (F value) and its corresponding P-value (Pr>F).

**Figure 4:**
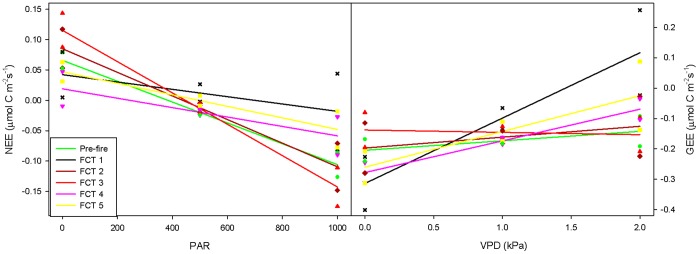
Interactive effect of PAR*FCT on NEE and VPD*FCT on GEE. Least square mean values by fire cycle time (FCT) (a) over a range of PAR for NEE, and (b) over a range of VPD for GEE. FCT 1 is the first 28 days following fire, FCT 2 and 3 are the next two 28 day periods, FCT 4 represents the next 140 days, and FCT 5 represents the next 224 days. To mitigate for autocorrelation PAR and NEE are 28 day means.

While NEE increased with PAR at all sites, this effect was significantly more dampened at the mesic site than at the other two sites (Table 3, Figure 3b). Fire also interacted with PAR to significantly affect NEE throughout the fire cycle. As would be expected, NEE increased with PAR regardless of FCT, but this relationship was weakest (i.e. flatter regression line) in the 28 days immediately following fire (Figure 4a, FCT = 1). Scheffe’s test indicated that there was a significant difference between the effect of PAR during FCT 1 versus that of pre-fire, with lower NEE in FCT 1 versus pre-fire levels (p = 0.0537). During FCT 2 and 3, PAR had a greater effect on carbon uptake than it did pre-fire, with a significant difference when comparing pre-fire with FCT 3 (Table 3, Figure 4a). During FCT 4 and 5, increasing PAR had a significantly smaller effect on NEE than it did pre-fire (i.e. flatter regression line; Table 3, Figure 4a).

R_eco_ increased significantly with T_air_ (p<0.0001) and significantly decreased with rising VPD (p<0.0001) (Table 3). There were no significant differences in R_eco_ over the fire cycle, but R_eco_ did significantly vary among sites (p = 0.0127). The xeric site had the lowest R_eco_ followed by the mesic and intermediate sites. The difference in R_eco_ between the xeric and intermediate sites was significantly different, but there was no significant difference found between the mesic site and xeric site.

In addition to PAR and T_air_, which were also significant in the model for NEE, VWC was a significant predictor in the model for GEE (Table 3). Not surprisingly, GEE significantly increased with PAR, T_air_ and VWC (p = 0.0115, p<0.0001, and p = 0.0008, respectively). There was also a significant interactive effect between FCT and VPD that was not identified in the model for NEE (p = 0.0018). With the exception of FCT 3, GEE decreased with VPD at all time periods (Figure 4b). At mean values of VPD (0.85 kPa), GEE was significantly lower in the thirty days following fire than in the thirty days before fire (Figure 4b). The effect of VPD on GEE was not significant during FCT 2, 3 and 5, but during FCT 4, increasing VPD resulted in significantly higher R_eco_. Increased VPD resulted in lower rates of GEE both before and after prescribed fire, but this effect was much stronger in the thirty days following fire than at any other time during the fire cycle (Figure 4b).

### Pre- and Post-fire Light and Temperature Response

**Figure 5:**
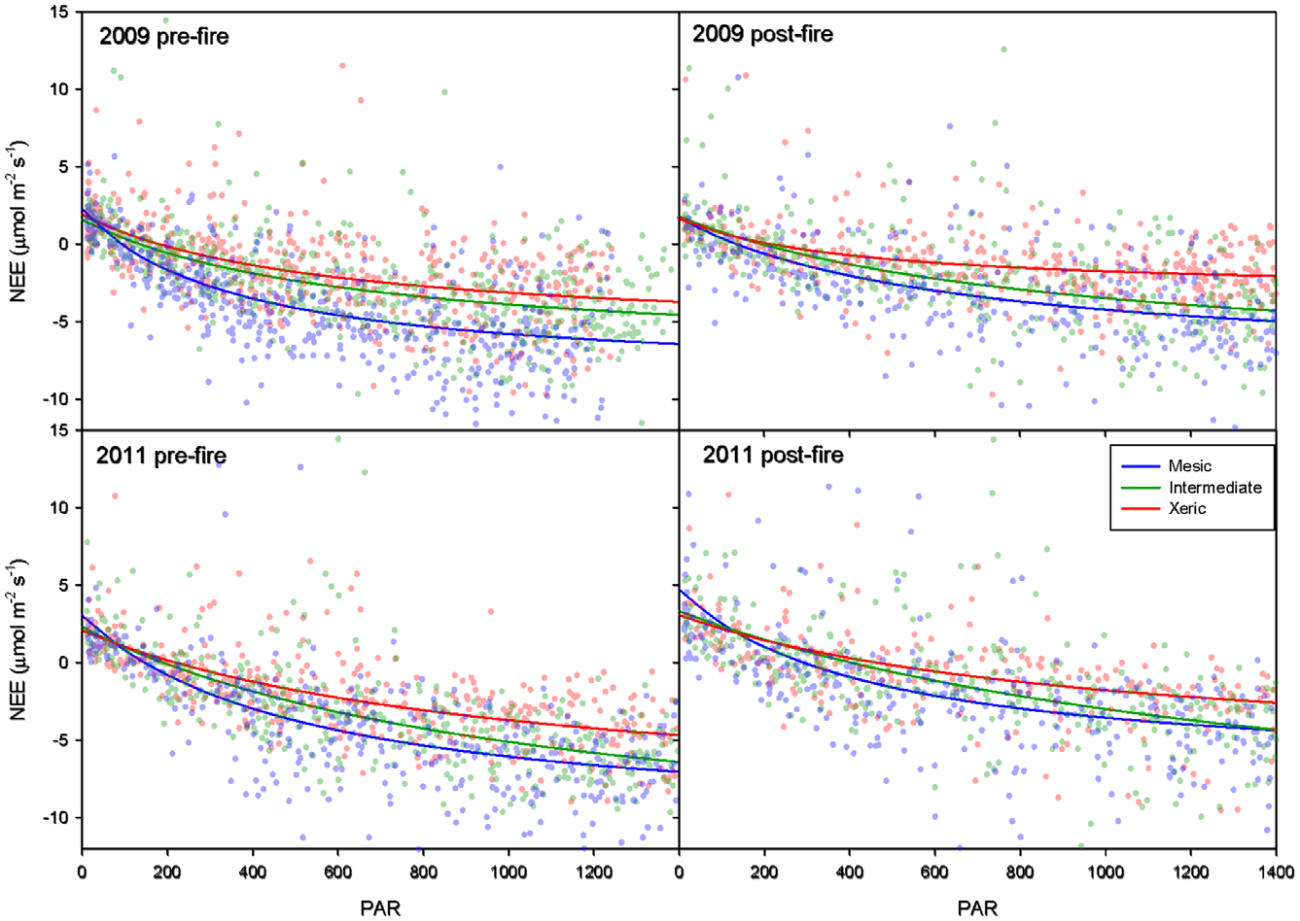
Representative pre- and post-fire light response curves. Representative light response curves pre- (left) and post-fire (right) in January 2009 (top) and March 2011 (bottom) at the xeric, intermediate and mesic sites (green, red and blue, respectively).

In general, fire decreased carbon uptake rates, and affected light response similarly in January 2009 and March 2011. Post-fire ecosystem light response curves were shallower when compared to pre-fire at all sites, indicating diminished carbon uptake (Figure 5). Among the sites, there were significant differences in estimated model parameters pre- and post-fire. Before fire in 2009 and 2011, apparent quantum yield (*α*) at the mesic site was significantly higher (i.e. higher carbon uptake) than at the other sites. Following fire in January 2009, *α* was significantly higher than pre-fire at the mesic and intermediate sites, and lower, but not significantly so, at the xeric site; however, there were no significant differences in *α* among the sites. In March 2011, *α* was not significantly affected by fire at any site. In 2009, *P_max_* was lower (i.e. lower maximum carbon uptake rate) post-fire at all three sites, but only significantly so at the xeric site (Figure 5). In March 2011, post-fire *P_max_* did not significantly differ from pre-fire values at any site (Figure 5). In 2009, the average value of *R_d_* at the mesic and intermediate sites decreased post-fire, while the average *R_d_* increased post-fire at the xeric site; however, these differences were not significant at any site.

**Figure 6:**
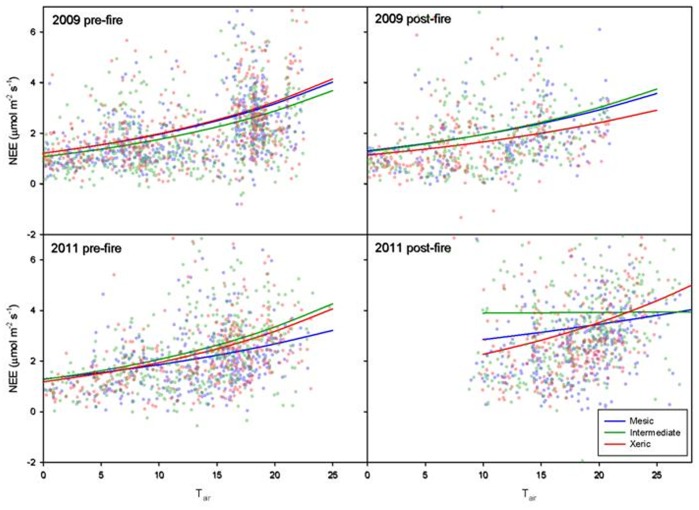
Representative pre- and post-fire temperature response curves. Representative nighttime temperature response curves pre- (left) and post-fire (right) in January 2009 (top) and March 2011 (bottom) at the xeric, intermediate and mesic sites (green, red and blue, respectively).

In 2009, fire reduced the response of nighttime respiration to temperature, especially at the xeric site (Figure 6); however, this effect was not significant. The effects of fire on nighttime NEE in March 2011 were more varied. Nighttime temperature response at the intermediate site was significantly affected by fire (Figure 6). Both *a*, the base respiration rate when air temperature is 0^o^C, and *b*, an empirical coefficient, were significantly different following fire at the intermediate site but not at the mesic and xeric sites. Post-fire in 2011, the base respiration rate was significantly higher at the intermediate site than at the mesic and xeric sites (Figure 6).

### Water Use Efficiency

**Figure 7:**
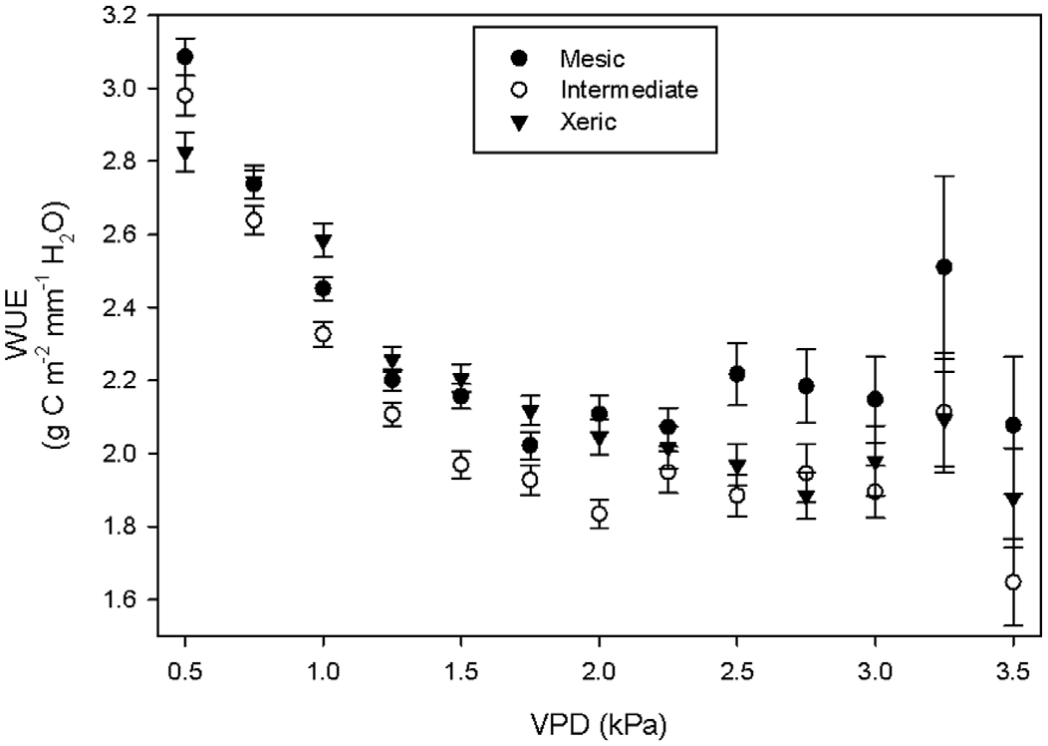
WUE versus VPD. WUE over a range of VPD values at the xeric, intermediate and mesic sites. WUE values were from the hours between 11∶00 and 15∶00 with PAR greater than 500 µmol m^−2^ s^−1^_._

ET, GEE and VPD were identified as variables that had a significant influence on WUE, and the influences of GEE and VPD were significantly different pre-and post-fire (Table 4). WUE was influenced more by ET than by GEE or VPD (Table 4), and decreased as ET increased. WUE did not significantly vary between the sites, but there were some significant differences in the way GEE and VPD affected WUE in the thirty days before and after fire. Increased GEE had a significantly smaller effect on WUE in the 28 days post-fire than during the 28 days pre-fire. Increasing VPD resulted in significantly greater WUE in the 28 days post-fire than it did in the 28 days pre-fire. There were no significant differences in ET pre- and post-fire nor among the sites; the only significant predictor of ET was VPD (Table 4), which showed a positive correlation with ET. We investigated WUE by VPD and site to gain insight into how stomatal dynamics affect WUE. WUE decreased from VPD values of 0.5 to approximately 2.0 kPa. As VPD values increased beyond 2.0 kPa, WUE became more constant, but dropped off precipitously at VPD of 3.5 kPa (Figure 7). At VPD values below 2.25 kPa, the intermediate site frequently had significantly lower WUE than the other two sites, while at VPD values above 2.25, the mesic site had higher WUE than the xeric and intermediate sites (Figure 7).

**Table 4:** Type III tests of fixed effects for the models of WUE and ET.

Effect	Num. DF	Den. DF	F Value	Pr>F
*Model of WUE*				
Post-fire	1	5	0.51	0.5065
ET	1	5	191.91	<.0001
GEE	1	5	92.43	0.0002
VPD	1	5	4.88	0.0782
GEE*Post-fire	1	5	12.18	0.0175
VPD*Post-fire	1	5	23.56	0.0047
*Model of ET*				
VPD	1	109.00	23.61	<.0001

WUE did not significantly differ among the sites, and ET did not significantly differ over the fire cycle or among the sites. Tables include for each effect, the degrees of freedom in the numerator (Num. DF), degrees of freedom in the denominator (Den. DF), and the value of the F statistic (F value) and its corresponding P-value (Pr>F).

## Discussion

This study shows the complexities that must be considered when studying the interactions between fire, soil water holding capacity, climate and carbon dynamics in a savanna ecosystem. Across our three sites environmental variables were near long-term means for the area [28] except for precipitation, which decreased each year of the study (Table 1). This lack of precipitation led to low VWC at all three sites, which strongly influenced the carbon dynamics and sequestration capacity in these ecosystems. Water limitation has previously been shown to strongly reduce above ground net primary production in longleaf pine ecosystems [26]. The three sites were comparable in all environmental variables except VWC, a characteristic we targeted in our site selection, and windspeed. Higher windspeed at the xeric site versus the mesic and intermediate sites was likely caused by differences in canopy structure; increased open canopy led to alterations in wind patterns caused by increased surface warming [58](Figure 1e). Alternatively, the higher windspeed at the xeric site could be a result of lower overstory density and trees of smaller stature, which impede wind flow at the measurement height less than the taller, more dense trees at the other sites [59], [60].

### Effect of Fire on Light and Temperature Response

Fire was associated with changes in vegetation and energy dynamics that affected how NEE responded to different light conditions. The decreased effect of PAR on CO_2_ uptake at all sites following fires in 2009 and 2011 (Figure 3b) was likely due to reductions in leaf area. Decreases in light response due to consumption of functional leaf area in the understory and leaf scorch in the overstory may have been mitigated by the timing of prescribed fires and longleaf pine canopy structure. Because understory grasses are dominated by C_4_ species, most leaf area in the herbaceous layer had yet to develop at the time of the fires. Similarly, hardwood shrubs also had not yet fully developed crowns. Thus, burns later in the growing season would increase the immediate impact on leaf area. Furthermore, longleaf pine crowns were above the height of the highest flames during this study and showed little to no damage. Differences in response of base respiration rates at zero PAR post-fire in 2009 and 2011 may have been caused by timing of fire. Mid-January is the deepest part of the dormant season, and physiological activity is at its lowest point annually, but by mid-March many understory plants are beginning to emerge from dormancy. Hence, respiration rates during the 28-day post-fire period in 2011 (from mid-March to mid-April) were likely augmented by increase autotrophic respiration associated with the flush of new leaves [61], [62].

Fire had more effects on nighttime temperature response following the fire in 2011 than it did following fire in 2009 (Figure 6). There were increases in *a* (base nighttime respiration rate when T_air_ is zero) at the mesic and intermediate sites following fire in 2011. The mesic site became less sensitive to changes in T_air_, and the intermediate site became nearly completely unaffected by T_air_, which suggests an abiotic source of CO_2_. Similar releases of carbon have been observed on ecosystems overlying karst topography [63], [64], [65], [66]. These studies suggest that interactions between organic acids in the soil and calcium carbonate in the soil and parent material may cause releases of CO_2_. There is, however, no consensus on how this interaction produces CO_2_, whether by dissolution of carbonate during wet conditions [65], [66], or by precipitation of carbonate as the soils dry [64]. At the sites in the current study, the soil and parent material are old and extensively weathered, so there may be little calcium carbonate available for reaction. Whatever the case, high carbon release at low temperature merits further investigation. Similar to light response, loss of leaf area likely caused the decrease in carbon release post-fire. Because soil respiration is tightly linked to recently assimilated carbohydrates [67], a decrease in carbon assimilation would likely result in reduced ecosystem respiration. Reduction in carbon release was greater in 2011 than in 2009, because by the time of the fire in March 2011, growth had already initiated. Differences in the effect of fire on nighttime respiration may have been affected by differing fire intensities at each site, and although we did not measure fire intensity, the potential for higher intensity fire certainly existed. The mesic and intermediate sites were more productive over the interval between the fires, which may have altered the fire intensity between the sites (Table 2, fuel consumption). Higher intensity fire would have a greater effect post fire ecosystem physiology [22].

### Environmental Effects

T_air_ and VPD had similar effects on R_eco_ at each site, but rates of R_eco_ were significantly different by site. Differences in R_eco_ between the sites were likely influenced by differences in productivity. Annual R_eco_ increased with GEE over the study period at each site. Recently assimilated carbohydrates fuel approximately 50% of the biological activity in soil [67], [68], and in many conifer forest ecosystems, R_eco_ tends to be dominated by soil respiration [43], [69], [70], [71]. In comparison to the xeric site, higher GEE at the mesic and intermediate sites likely resulted in increased R_eco_. T_air_ is highly correlated with soil temperature, so the effect of T_air_ on R_eco_ likely reflects the influence of changing soil temperature on root and microbial soil respiration. As VPD increases, reduced stomatal apertures restrict diffusion and lower rates of photosynthesis, which presumably decreases carbohydrates available for respiration below ground.

GEE was significantly affected by PAR, T_air­_ and VWC, and the interaction of VPD and FCT. Increases in GEE with PAR and T_air_ reflect diurnal changes. PAR drives photosynthesis and both PAR and T­_air_ peak during the day and reach minima during the night. Increased GEE with increasing VWC reflected differences in VWC between sites. Previous studies along this soil moisture gradient have also found increased GEE with increased soil water holding capacity [26], [30], [72]. With the exception of FCT 2 (the 28 day period starting 56 days after fire), GEE decreased with VPD as time since fire increased (Figure 4b). Average VPD during FCT 2 was the lowest of any other FCT class (data not shown), which may explain why increasing VPD had so little effect on GEE over that time period. Increasing VPD had a stronger, negative effect on GEE in the 28 days following fire (FCT 0) than at any other time during the fire cycle (Figure 4b). The strong relationship between VPD and GEE during FCT 0 may be caused by reduced fine root biomass and its effects on energy dynamics. Because access to soil water is limited post-fire, stomata in surviving vegetation may be more sensitive to increases in VPD [73], [74], [75].

PAR, T_air_, VWC, VPD, and windspeed were the most critical variables driving NEE in this work, and have previously been reported to impact carbon dynamics in longleaf pine ecosystems [72]. The effect of PAR on NEE varied significantly between sites and throughout the fire cycle. The most conspicuous effect of fire is the destruction of leaves and stems above ground. In the 28 days immediately following fire (FCT 0), this reduction in photosynthetic area likely accounted for the reduction in NEE, and a dampening of the effect of PAR on NEE (Figure 4a). As the ecosystem continued to recover (FCT 2 and 3, approximately the 2^nd^ and 3^rd^ months following fire), NEE was more strongly affected by changes in PAR. Increased uptake at high light levels may reflect a post-fire increase in plant nutrients. Low intensity fire causes a short term increase in soil nutrient availability [76], [77], [78], and leaves re-grown following fire are often enriched in phosphorus and nitrogen and may attain higher carbon assimilation rates than older, pre-fire leaves [78], [79]. Furthermore, there can be an increase in the total amount of functional leaf area and hence an increase in total photosynthetic machinery in the ecosystem [78]. Increased release of carbon at low light levels indicates an increase in respiration. Metabolic respiration increases as leaves and other plant parts lost during fire are replaced [12]. Furthermore, as discussed above, soil respiration may be dependent on recently assimilated carbohydrates, so the increase in carbon uptake at high light levels may also result in an increase in carbon release at lower light levels.

Rates of carbon uptake increased with PAR at all three sites, but the strength of this relationship was diminished at the mesic site (Figure 3b). Other studies contrasting carbon dynamics at these same mesic and xeric sites found that water availability had a strong influence on NEE [72], [80]. Trees at the mesic and xeric sites have developed differences in hydraulic architecture that affect how they respond to drought [81]. Trees at the mesic site avoid drought while xeric site trees tolerate drought [80]. During drought, the mesic site avoided damage due to water stress by reducing LAI by 30% compared to non-drought periods. LAI at the xeric site was unaffected by drought, and the trees simply tolerated the lack of moisture. Similarly, productivity was 30% higher at the mesic site compared to the xeric site during non-drought years, but equal between the sites during drought [80]. The differences in the effect of increasing PAR among sites suggests that higher productivity at the mesic site is due to lower respiration rates rather than to higher rates of carbon uptake. Our results support Wright et al. [80] and show that, as drought stress increased, NEE response to light decreased at the mesic site but increased at the other two sites (Figure 5). There may be further adaptations to site differences in water availability that affect how trees respond to drought. Differences in hydraulic architecture allowed trees on the xeric site to maintain similar and sometimes higher stomatal conductance than trees on the mesic site [81]. Furthermore, trees on the xeric site were shorter of stature and had higher root-to-leaf area ratios, which made them better adapted to cope with lower soil water availability [81].

Differences in the effect of windspeed on NEE were likely caused by seasonal changes, phenology, and higher average windspeed at the xeric site than at mesic and intermediate sites. During times of low windspeed, the air within the canopy does not mix well with the free atmosphere above, and CO_2_ often builds up beneath the canopy [82], [83], [84]. As windspeeds increase and the atmosphere becomes well mixed, the built up CO_2_ is released, which has been shown in a number of studies [83], [84]. Because the canopy is more open and windspeeds are generally higher at the xeric site (Figure 1), there are fewer and smaller build-ups of CO_2_ within the plant canopy when atmospheric mixing is poor and smaller releases of CO_2_ when the atmosphere becomes mixed. At the study sites, average windspeeds are typically higher in the winter and spring, and lower during the summer and fall (Figure 1e). Because of its low overstory density and high proportion of C_4_ grasses relative to the other two sites, the xeric site is able to be more productive in the hotter and calmer summer months (Figure 2). Conversely, the high proportion of C_4_ grasses at the xeric site are less productive in the cooler and windier springtime months.

### Annual Carbon Balance

Low net carbon uptake at the mesic site and carbon release at the xeric and intermediate sites in comparison to fuel consumption shows how longleaf pine ecosystems are adapted to frequent fire and drought. Low or no net uptake indicates that the carbon consumed during the fires were assimilated prior to the preceding fire. Longleaf pine trees and other plants in these ecosystems recover quickly from fire in part due to carbohydrates stored in their roots [15]. Carbohydrates stored in the roots may have applications for the plants outside of fast post-fire recovery. Drought causes long-term reductions in carbon uptake, but growth in longleaf pine can be supported for extended periods by carbohydrates stored in the roots [85].

Annual variation between the sites in the ratio of R_eco_/GEE was likely affected by differences in nutrient availability. In longleaf pine ecosystems, nitrogen mineralization decreases with increased soil moisture availability [86]; however, increased nitrogen availability does not result in increased above ground primary productivity in longleaf pine ecosystems [26]. The effects of increased nutrient availability were likely manifested more in differences in R_eco_. Valentini et al. [87] found that NEE was governed more by respiration than GPP over a latitudinal gradient in European forests. In this work, productivity in longleaf pine ecosystems along our soil moisture gradient appeared to be governed more by differences in respiration.

High R_eco_ relative to GEE during the first year of the study at the xeric site may have resulted from increased soil respiration when soil moisture increased following drought. During much of the two years prior to this study, our study sites were under extreme drought conditions [88]. As annual precipitation returned to normal, soil respiration may have been abnormally high due to a phenomenon known as the “Birch Effect” where re-wetting of dry soils results in an increase in soil respiration [89]. Following summer drought in an oak savanna in Portugal, the Birch Effect resulted in a net ecosystem loss of 248 g C m^−2^ over three months [90]. Further, GPP was not significantly affected by the increased soil moisture, and did not offset increased soil respiration [90]. The magnitude of soil respiration increases with the degree and duration of the preceding dry period, as well as with the magnitude of the relative change of soil water content when the soil is re-wetted [91]. In the current study cumulative NEE over the first year following drought was highest at the xeric site followed by the intermediate and mesic sites. Higher average soil moisture at the mesic and intermediate sites likely prevented increases in soil respiration due to the Birch effect.

### Water Use Efficiency

Over the study period in general, WUE increased with GEE and decreased with rising ET and VPD, but those relationships were affected by fire. Reduction in the effect of GEE on WUE following fire was likely caused by loss of leaf area and a resultant decrease in carbon uptake (Figure 7). The reversal in the relationship between WUE and VPD following fire may have been consistent with reduced ET rates associated with increases in VPD. The effect of water stress on stomatal response to rising VPD likely caused reductions in ET. Decreased stomatal apertures restrict diffusion of H_2_O about 1.6 times more than CO_2_ [39], which results in greater WUE. As discussed above, there is a short-term increase in nutrient availability in the soil following low intensity fire [76], [77], [78]. Increases in nutrient availability have been shown to decrease stomatal conductance in similar southern pine species [92], which may represent another mechanism that decreases ET and leads to increases in WUE.

### Study Limitations

This study quantifies carbon dynamics over three years using data from three sites along an edaphic moisture gradient. The experimental design of this research could be enhanced by replicating the design in multiple similar ecosystems. Lack of replication has been widely recognized as a limitation in large-scale ecosystem experiments of this kind, where replication can be impossible due to funding constraints – or due to the lack of adequate replicates available when large systems are studied. Appropriate scaling of experimental units, however, has been viewed by some ecologists as more important than replication [93]. Due to the large footprint of EC measurements and unique characteristics of the systems of study, the three sites chosen as experimental units in this study represent the spatial and temporal scale relevant for predictions in this system. Moreover, the sites serve as a proxy for possible change in soil water availability caused by predicted climate change and allow for the testing of alternative hypotheses. On the other hand, lack of true replication does limit the scope of inference of the results from the study, and provides motivation for ongoing research in this area.

### Conclusion

The findings of this study advance our understanding of the complex interactions that occur between fire, soil water availability and carbon dynamics for longleaf pine ecosystems. However, there is still a considerable amount of knowledge to be gained in regards to the long-term carbon sequestration capacity of this ecosystem, especially in the face of changing precipitation patterns, which are a prediction of climate change. Thus we argue that the scientific community should endeavor to study these interactions over decadal scales, which would include additional fire cycles and a larger variance in environmental conditions. By taking on long-term studies we will be able to draw greater insight on how longleaf pine systems and savanna ecosystems respond to changes in water availability and fire. This will be key in determining the future contribution of savanna ecosystem to the global carbon budget.

## Supporting Information

Table S1 **Distribution of parameters from NEE bootstrap simulations.** LCL = lower limit of 90% confidence region, UCL = upper limit of 90% confidence region.
(DOCX)

Table S2 **Distribution of parameters from Reco bootstrap simulations.** LCL = lower limit of 90% confidence region, UCL = upper limit of 90% confidence region.
(DOCX)
