# Supplementary material for: Cyclic Occurrence of Fire and Its Role in Carbon Dynamics along an Edaphic Moisture Gradient in Longleaf Pine Ecosystems
Source: PLoS One. 2013 Jan 15;8(1):e54045. doi: 10.1371/journal.pone.0054045 (PMC3545999; doi:10.1371/journal.pone.0054045)
Supplement: Table S1 — Distribution of parameters from NEE bootstrap simulations. LCL = lower limit of 90% confidence region, UCL = upper limit of 90% confidence region. (DOCX) [file pone.0054045.s001.docx]

Table S.1. Distribution of parameters from NEE bootstrap simulations. LCL = lower limit of 90% confidence region, UCL = upper limit of 90% confidence region.

|  |  |  | *α* | | | *P_max_* | | | *R_eco_* | | |
| --- | --- | --- | --- | --- | --- | --- | --- | --- | --- | --- | --- |
| Site | Year | Month | Estim. Param. | LCL | UCL | Estim. Param. | LCL | UCL | Estim. Param. | LCL | UCL |
| Mesic | 2008 | 10 | -0.033 | -0.058 | -0.013 | -19.145 | -34.951 | -14.987 | 3.350 | 0.786 | 5.452 |
|  |  | 11 | -0.031 | -0.041 | -0.024 | -12.263 | -13.451 | -11.427 | 2.831 | 2.378 | 3.424 |
|  |  | 12 | -0.038 | -0.049 | -0.029 | -12.373 | -13.462 | -11.543 | 2.483 | 1.998 | 3.029 |
|  | 2009 | 1 | -0.029 | -0.042 | -0.021 | -9.004 | -10.007 | -8.216 | 1.946 | 1.443 | 2.503 |
|  |  | 2 | -0.016 | -0.020 | -0.012 | -15.115 | -17.807 | -13.549 | 2.524 | 1.973 | 3.119 |
|  |  | 3 | -0.014 | -0.019 | -0.010 | -17.704 | -22.487 | -15.339 | 2.074 | 1.485 | 2.837 |
|  |  | 4 | -0.029 | -0.036 | -0.024 | -16.320 | -17.357 | -15.480 | 4.033 | 3.403 | 4.835 |
|  |  | 5 | -0.048 | -0.058 | -0.040 | -22.720 | -23.922 | -21.698 | 5.953 | 5.083 | 6.787 |
|  |  | 6 | -0.053 | -0.071 | -0.041 | -21.893 | -23.671 | -20.539 | 6.749 | 5.389 | 8.440 |
|  |  | 7 | -0.053 | -0.070 | -0.042 | -26.503 | -28.079 | -25.026 | 9.014 | 7.570 | 10.645 |
|  |  | 8 | -0.043 | -0.054 | -0.034 | -26.159 | -28.180 | -24.491 | 6.849 | 5.687 | 8.060 |
|  |  | 9 | -0.051 | -0.062 | -0.043 | -27.214 | -28.835 | -25.812 | 7.835 | 6.762 | 9.084 |
|  |  | 10 | -0.038 | -0.045 | -0.032 | -23.980 | -25.777 | -22.540 | 5.358 | 4.539 | 6.196 |
|  |  | 11 | -0.034 | -0.050 | -0.023 | -20.594 | -23.662 | -18.903 | 4.035 | 2.690 | 5.640 |
|  |  | 12 | -0.030 | -0.038 | -0.025 | -18.028 | -20.321 | -16.473 | 2.520 | 1.867 | 3.332 |
|  | 2010 | 1 | -0.035 | -0.071 | -0.017 | -6.626 | -7.848 | -5.849 | 2.271 | 1.372 | 3.619 |
|  |  | 2 | -0.043 | -0.057 | -0.032 | -9.374 | -10.308 | -8.622 | 2.568 | 1.879 | 3.447 |
|  |  | 3 | -0.018 | -0.023 | -0.015 | -12.618 | -13.642 | -11.729 | 2.255 | 1.760 | 2.912 |
|  |  | 4 | -0.022 | -0.027 | -0.017 | -21.071 | -23.648 | -19.518 | 4.192 | 3.530 | 4.913 |
|  |  | 5 | -0.033 | -0.040 | -0.028 | -27.485 | -29.497 | -25.714 | 6.550 | 5.611 | 7.621 |
|  |  | 6 | -0.034 | -0.045 | -0.026 | -23.459 | -25.502 | -21.845 | 6.794 | 5.537 | 8.279 |
|  |  | 7 | -0.036 | -0.047 | -0.028 | -18.084 | -19.619 | -16.857 | 5.791 | 5.030 | 6.666 |
|  |  | 8 | -0.047 | -0.062 | -0.036 | -19.948 | -21.536 | -18.456 | 7.236 | 6.164 | 8.518 |
|  |  | 9 | -0.025 | -0.035 | -0.020 | -22.525 | -25.433 | -20.201 | 5.555 | 4.461 | 7.146 |
|  |  | 10 | -0.015 | -0.023 | -0.011 | -15.337 | -19.281 | -13.548 | 3.176 | 2.275 | 4.199 |
|  |  | 11 | -0.034 | -0.056 | -0.023 | -14.435 | -16.551 | -12.869 | 4.238 | 2.884 | 6.174 |
|  |  | 12 | -0.010 | -0.014 | -0.007 | -12.206 | -19.315 | -9.211 | 1.168 | 0.808 | 1.519 |
|  | 2011 | 1 | -0.025 | -0.041 | -0.015 | -9.668 | -11.379 | -8.366 | 2.586 | 1.402 | 4.079 |
|  |  | 2 | -0.021 | -0.028 | -0.016 | -12.994 | -14.799 | -11.929 | 2.359 | 1.901 | 2.910 |
|  |  | 3 | -0.044 | -0.066 | -0.031 | -12.196 | -14.025 | -10.893 | 4.917 | 3.769 | 6.646 |
|  |  | 4 | -0.022 | -0.030 | -0.018 | -16.193 | -17.749 | -15.087 | 4.259 | 3.554 | 5.128 |
|  |  | 5 | -0.025 | -0.032 | -0.020 | -13.423 | -14.547 | -12.556 | 3.715 | 3.263 | 4.237 |
|  |  | 6 | -0.035 | -0.049 | -0.026 | -9.091 | -9.951 | -8.264 | 4.435 | 3.771 | 5.207 |
|  |  | 7 | -0.026 | -0.034 | -0.020 | -16.812 | -18.620 | -15.627 | 5.841 | 5.048 | 6.918 |
|  |  | 8 | -0.030 | -0.049 | -0.021 | -19.532 | -22.243 | -17.746 | 6.467 | 4.997 | 8.967 |
|  |  | 9 | -0.032 | -0.048 | -0.021 | -11.631 | -13.145 | -10.540 | 3.120 | 2.232 | 4.168 |
|  |  | 10 | -0.021 | -0.035 | -0.011 | -11.023 | -13.873 | -9.626 | 3.379 | 2.409 | 4.580 |
| Intermediate | 2008 | 10 | -0.012 | -0.018 | -0.009 | -49.784 | -220.800 | -30.442 | 2.273 | 1.091 | 3.846 |
|  |  | 11 | -0.017 | -0.023 | -0.013 | -34.354 | -51.631 | -27.689 | 3.774 | 2.917 | 4.688 |
|  |  | 12 | -0.012 | -0.019 | -0.007 | -25.952 | -503.900 | -15.661 | 2.078 | 1.252 | 3.115 |
|  | 2009 | 1 | -0.014 | -0.019 | -0.010 | -8.835 | -10.217 | -7.892 | 1.658 | 1.291 | 2.028 |
|  |  | 2 | -0.013 | -0.019 | -0.009 | -13.893 | -17.551 | -12.175 | 2.681 | 1.979 | 3.511 |
|  |  | 3 | -0.023 | -0.036 | -0.016 | -14.181 | -15.745 | -13.177 | 4.020 | 3.102 | 5.273 |
|  |  | 4 | -0.028 | -0.034 | -0.023 | -22.162 | -23.635 | -20.986 | 4.750 | 3.939 | 5.685 |
|  |  | 5 | -0.038 | -0.044 | -0.032 | -25.571 | -27.444 | -24.105 | 5.312 | 4.760 | 5.897 |
|  |  | 6 | -0.037 | -0.047 | -0.029 | -20.198 | -21.721 | -18.940 | 6.504 | 5.740 | 7.402 |
|  |  | 7 | -0.052 | -0.072 | -0.040 | -21.633 | -23.282 | -20.317 | 8.325 | 6.979 | 9.959 |
|  |  | 8 | -0.038 | -0.051 | -0.029 | -27.419 | -30.541 | -25.152 | 8.122 | 6.759 | 9.786 |
|  |  | 9 | -0.041 | -0.055 | -0.032 | -25.197 | -27.386 | -23.676 | 7.514 | 6.395 | 9.014 |
|  |  | 10 | -0.033 | -0.044 | -0.025 | -21.642 | -23.596 | -20.288 | 5.984 | 4.963 | 7.286 |
|  |  | 11 | -0.022 | -0.033 | -0.015 | -17.070 | -20.836 | -15.580 | 3.722 | 2.699 | 4.846 |
|  |  | 12 | -0.025 | -0.029 | -0.021 | -15.218 | -16.848 | -14.025 | 2.333 | 1.960 | 2.708 |
|  | 2010 | 1 | -0.027 | -0.043 | -0.014 | -6.197 | -7.050 | -5.668 | 1.915 | 1.196 | 2.577 |
|  |  | 2 | -0.027 | -0.039 | -0.020 | -9.680 | -10.529 | -9.071 | 2.394 | 1.779 | 3.259 |
|  |  | 3 | -0.018 | -0.024 | -0.013 | -11.436 | -12.448 | -10.711 | 2.847 | 2.272 | 3.543 |
|  |  | 4 | -0.023 | -0.029 | -0.018 | -21.070 | -23.591 | -19.345 | 5.016 | 4.217 | 5.892 |
|  |  | 5 | -0.033 | -0.040 | -0.028 | -28.575 | -30.834 | -26.660 | 6.067 | 5.379 | 6.867 |
|  |  | 6 | -0.033 | -0.041 | -0.026 | -27.717 | -30.697 | -25.592 | 7.444 | 6.383 | 8.589 |
|  |  | 7 | -0.032 | -0.039 | -0.025 | -22.308 | -24.559 | -20.689 | 7.112 | 6.082 | 8.212 |
|  |  | 8 | -0.033 | -0.042 | -0.028 | -23.958 | -26.546 | -22.100 | 7.812 | 6.979 | 8.993 |
|  |  | 9 | -0.015 | -0.022 | -0.011 | -17.539 | -21.991 | -15.100 | 4.268 | 3.421 | 5.435 |
|  |  | 10 | -0.011 | -0.019 | -0.007 | -15.473 | -22.699 | -13.184 | 3.531 | 2.398 | 5.026 |
|  |  | 11 | -0.008 | -0.012 | -0.005 | -26.094 | -468.600 | -15.776 | 2.155 | 1.424 | 3.104 |
|  |  | 12 | -0.012 | -0.023 | -0.008 | -8.734 | -12.289 | -7.310 | 1.792 | 1.198 | 2.616 |
|  | 2011 | 1 | -0.017 | -0.026 | -0.011 | -11.286 | -13.145 | -10.250 | 2.390 | 1.531 | 3.341 |
|  |  | 2 | -0.012 | -0.019 | -0.008 | -15.448 | -21.748 | -12.993 | 1.968 | 1.340 | 2.741 |
|  |  | 3 | -0.014 | -0.019 | -0.010 | -13.420 | -16.474 | -11.902 | 2.783 | 2.230 | 3.414 |
|  |  | 4 | -0.026 | -0.037 | -0.019 | -19.642 | -21.892 | -18.117 | 6.122 | 4.745 | 7.850 |
|  |  | 5 | -0.025 | -0.031 | -0.020 | -17.522 | -18.893 | -16.498 | 4.274 | 3.678 | 4.991 |
|  |  | 6 | -0.035 | -0.054 | -0.024 | -7.363 | -8.282 | -6.691 | 4.070 | 3.471 | 4.945 |
|  |  | 7 | -0.026 | -0.033 | -0.021 | -24.129 | -27.319 | -22.118 | 6.307 | 5.647 | 7.170 |
|  |  | 8 | -0.028 | -0.033 | -0.023 | -29.277 | -32.355 | -27.033 | 6.610 | 5.583 | 7.681 |
|  |  | 9 | -0.032 | -0.042 | -0.024 | -23.067 | -26.056 | -21.192 | 5.427 | 4.468 | 6.628 |
|  |  | 10 | -0.019 | -0.029 | -0.012 | -15.071 | -19.722 | -13.032 | 3.559 | 2.720 | 4.667 |
| Xeric | 2008 | 12 | -0.012 | -0.021 | -0.007 | -9.206 | -17.156 | -7.552 | 1.474 | 0.827 | 2.214 |
|  | 2009 | 1 | -0.015 | -0.025 | -0.010 | -6.185 | -7.355 | -5.556 | 1.989 | 1.575 | 2.488 |
|  |  | 2 | -0.012 | -0.022 | -0.007 | -6.682 | -8.264 | -5.945 | 2.009 | 1.432 | 2.828 |
|  |  | 3 | -0.014 | -0.019 | -0.011 | -10.019 | -11.631 | -8.866 | 3.339 | 2.802 | 3.994 |
|  |  | 4 | -0.031 | -0.043 | -0.023 | -13.017 | -14.357 | -12.022 | 4.338 | 3.525 | 5.413 |
|  |  | 5 | -0.054 | -0.065 | -0.046 | -22.861 | -24.075 | -21.812 | 6.631 | 5.834 | 7.523 |
|  |  | 6 | -0.054 | -0.074 | -0.042 | -20.565 | -22.327 | -19.269 | 7.098 | 6.046 | 8.747 |
|  |  | 7 | -0.047 | -0.059 | -0.038 | -22.202 | -23.862 | -20.876 | 8.322 | 7.136 | 9.710 |
|  |  | 8 | -0.039 | -0.056 | -0.029 | -27.526 | -30.375 | -25.299 | 7.648 | 5.851 | 10.090 |
|  |  | 9 | -0.038 | -0.049 | -0.030 | -21.440 | -22.988 | -20.190 | 6.338 | 5.251 | 7.672 |
|  |  | 10 | -0.036 | -0.044 | -0.029 | -20.102 | -21.861 | -18.789 | 5.611 | 4.731 | 6.608 |
|  |  | 11 | -0.018 | -0.030 | -0.011 | -15.196 | -21.185 | -13.117 | 2.823 | 1.925 | 3.966 |
|  |  | 12 | -0.022 | -0.032 | -0.016 | -10.678 | -12.221 | -9.637 | 2.511 | 1.962 | 3.334 |
|  | 2010 | 1 | -0.024 | -0.045 | -0.013 | -4.069 | -4.715 | -3.576 | 1.811 | 1.248 | 2.485 |
|  |  | 2 | -0.024 | -0.032 | -0.019 | -6.267 | -6.739 | -5.863 | 1.827 | 1.494 | 2.205 |
|  |  | 3 | -0.005 | -0.008 | -0.004 | -15.577 | -36.637 | -10.225 | 1.053 | 0.748 | 1.437 |
|  |  | 4 | -0.009 | -0.014 | -0.006 | -25.195 | -68.627 | -17.565 | 2.483 | 1.640 | 3.554 |
|  |  | 5 | -0.032 | -0.043 | -0.024 | -28.856 | -32.571 | -26.431 | 5.123 | 3.916 | 6.699 |
|  |  | 6 | -0.032 | -0.047 | -0.023 | -18.480 | -20.767 | -16.790 | 4.730 | 3.512 | 6.422 |
|  |  | 7 | -0.033 | -0.044 | -0.026 | -18.341 | -20.214 | -16.973 | 6.246 | 5.387 | 7.359 |
|  |  | 8 | -0.042 | -0.055 | -0.034 | -18.996 | -20.625 | -17.680 | 6.748 | 5.905 | 7.781 |
|  |  | 9 | -0.017 | -0.021 | -0.014 | -16.932 | -19.568 | -14.879 | 3.902 | 3.209 | 4.747 |
|  |  | 10 | -0.017 | -0.024 | -0.012 | -15.052 | -17.650 | -13.482 | 3.576 | 2.675 | 4.764 |
|  |  | 11 | -0.025 | -0.049 | -0.015 | -13.034 | -14.957 | -11.839 | 3.816 | 2.566 | 5.900 |
|  |  | 12 | -0.006 | -0.009 | -0.005 | -15.328 | -48.025 | -9.227 | 0.981 | 0.752 | 1.238 |
|  | 2011 | 1 | -0.016 | -0.025 | -0.010 | -6.130 | -7.020 | -5.575 | 1.431 | 0.943 | 2.054 |
|  |  | 2 | -0.010 | -0.014 | -0.006 | -10.806 | -16.781 | -9.211 | 1.518 | 0.931 | 2.151 |
|  |  | 3 | -0.021 | -0.032 | -0.014 | -8.389 | -9.433 | -7.730 | 3.456 | 2.833 | 4.247 |
|  |  | 4 | -0.025 | -0.032 | -0.020 | -20.792 | -23.633 | -18.763 | 5.250 | 4.472 | 6.125 |
|  |  | 5 | -0.030 | -0.037 | -0.024 | -16.927 | -18.226 | -15.835 | 4.813 | 4.203 | 5.595 |
|  |  | 6 | -0.023 | -0.031 | -0.017 | -11.499 | -12.640 | -10.783 | 3.766 | 3.335 | 4.391 |
|  |  | 7 | -0.027 | -0.036 | -0.021 | -20.175 | -22.380 | -18.444 | 6.117 | 5.195 | 7.403 |
|  |  | 8 | -0.027 | -0.036 | -0.020 | -19.797 | -22.096 | -17.893 | 5.761 | 4.622 | 7.331 |
|  |  | 9 | -0.033 | -0.043 | -0.025 | -17.581 | -19.380 | -16.370 | 4.555 | 3.730 | 5.506 |
|  |  | 10 | -0.016 | -0.027 | -0.010 | -10.146 | -12.434 | -9.189 | 2.028 | 1.357 | 2.844 |
|  | Annual (yr 1) | | -0.020 | -0.021 | -0.018 | -18.660 | -19.666 | -17.876 | 3.729 | 3.462 | 4.002 |

Table S.2. Distribution of parameters from R_eco_ bootstrap simulations. LCL = lower limit of 90% confidence region, UCL = upper limit of 90% confidence region.

|  |  |  | *R_0_* | | | *B* | | |
| --- | --- | --- | --- | --- | --- | --- | --- | --- |
| Site | Year | Month | Estim. Param. | LCL | UCL | Estim. Param. | LCL | UCL |
| Mesic | 2008 | 10 | 1.455 | 0.0898 | 4.7046 | 0.038 | -0.0219 | 0.1851 |
|  |  | 11 | 1.318 | 1.0986 | 1.5655 | 0.048 | 0.0352 | 0.0613 |
|  |  | 12 | 1.138 | 1.0044 | 1.2908 | 0.051 | 0.0425 | 0.0595 |
|  | 2009 | 1 | 1.180 | 1.0628 | 1.3072 | 0.046 | 0.0387 | 0.0532 |
|  |  | 2 | 1.148 | 0.8394 | 1.4637 | 0.050 | 0.0315 | 0.0734 |
|  |  | 3 | 1.315 | 0.9939 | 1.6134 | 0.052 | 0.039 | 0.0681 |
|  |  | 4 | 1.093 | 0.8019 | 1.411 | 0.065 | 0.0495 | 0.0828 |
|  |  | 5 | 2.804 | 1.7013 | 4.098 | 0.029 | 0.01 | 0.0523 |
|  |  | 6 | 3.349 | 1.5979 | 6.9929 | 0.022 | -0.00697 | 0.0514 |
|  |  | 8 | 1.174 | 0.1816 | 6.2642 | 0.077 | 0.00763 | 0.1547 |
|  |  | 9 | 5.713 | 2.6984 | 13.1515 | 0.007 | -0.0295 | 0.0393 |
|  |  | 10 | 1.828 | 1.3851 | 2.2527 | 0.052 | 0.0421 | 0.0659 |
|  |  | 11 | 2.447 | 1.4905 | 3.5475 | 0.032 | 0.00282 | 0.0707 |
|  |  | 12 | 1.587 | 1.2694 | 1.9947 | 0.037 | 0.0171 | 0.0561 |
|  | 2010 | 1 | 0.761 | 0.7055 | 0.8245 | 0.069 | 0.0625 | 0.0761 |
|  |  | 2 | 0.816 | 0.7152 | 0.912 | 0.068 | 0.0578 | 0.081 |
|  |  | 3 | 1.177 | 0.9119 | 1.4985 | 0.056 | 0.0413 | 0.0718 |
|  |  | 4 | 1.605 | 1.0159 | 2.593 | 0.045 | 0.0201 | 0.0688 |
|  |  | 5 | 3.049 | 1.4617 | 5.3043 | 0.026 | 0.00108 | 0.0609 |
|  |  | 11 | 1.162 | 0.6874 | 1.5557 | 0.059 | 0.0391 | 0.0946 |
|  |  | 12 | 0.876 | 0.7597 | 1.0033 | 0.048 | 0.0415 | 0.0552 |
|  | 2011 | 1 | 0.577 | 0.2639 | 0.8894 | 0.115 | 0.0762 | 0.1802 |
|  |  | 2 | 1.158 | 0.9909 | 1.3914 | 0.044 | 0.0321 | 0.0529 |
|  |  | 3 | 1.559 | 1.1204 | 2.0335 | 0.037 | 0.0206 | 0.0575 |
|  |  | 5 | 1.955 | 1.4921 | 2.4843 | 0.031 | 0.02 | 0.0429 |
|  |  | 8 | 2.247 | 0.3574 | 13.3571 | 0.038 | -0.0314 | 0.1085 |
|  |  | 9 | 3.678 | 2.4929 | 5.3538 | 0.001 | -0.0163 | 0.0183 |
|  |  | 10 | 2.293 | 1.6053 | 3.0127 | 0.001 | -0.0172 | 0.0221 |
|  | Annual (yr 1) | | 1.009 | 0.9247 | 1.1034 | 0.075 | 0.0701 | 0.0791 |
|  | Annual (yr 2) | | 1.259 | 1.1898 | 1.3343 | 0.060 | 0.0567 | 0.0622 |
|  | Annual (yr 3) | | 1.269 | 1.1642 | 1.3713 | 0.050 | 0.0461 | 0.054 |
| Intermediate | 2008 | 10 | 3.027 | 2.4654 | 3.7045 | 0.032 | 0.0167 | 0.046 |
|  |  | 11 | 2.910 | 2.4731 | 3.3384 | 0.023 | 0.0129 | 0.035 |
|  |  | 12 | 1.723 | 1.4396 | 2.0875 | 0.019 | 0.00693 | 0.0308 |
|  | 2009 | 1 | 1.132 | 1.014 | 1.2478 | 0.054 | 0.0465 | 0.0616 |
|  |  | 2 | 1.651 | 1.4345 | 1.8873 | 0.023 | 0.0127 | 0.0322 |
|  |  | 3 | 2.297 | 1.8906 | 2.8213 | 0.027 | 0.0163 | 0.0374 |
|  |  | 4 | 1.317 | 0.9585 | 1.6626 | 0.058 | 0.0442 | 0.0758 |
|  |  | 9 | 6.968 | 3.8577 | 12.1759 | 0.000 | -0.023 | 0.0248 |
|  |  | 10 | 2.549 | 2.1673 | 2.9417 | 0.035 | 0.027 | 0.0429 |
|  |  | 12 | 1.456 | 1.2858 | 1.6456 | 0.048 | 0.0385 | 0.0567 |
|  | 2010 | 1 | 1.001 | 0.8107 | 1.1619 | 0.062 | 0.0491 | 0.0812 |
|  |  | 2 | 1.252 | 1.1201 | 1.3708 | 0.056 | 0.0466 | 0.0657 |
|  |  | 3 | 1.016 | 0.8623 | 1.1878 | 0.075 | 0.0648 | 0.087 |
|  |  | 5 | 3.574 | 2.2085 | 5.4983 | 0.020 | 0.00115 | 0.0417 |
|  |  | 8 | 6.601 | 2.4918 | 16.0456 | 0.002 | -0.0332 | 0.0405 |
|  |  | 11 | 2.408 | 1.9086 | 3.0429 | 0.006 | -0.00742 | 0.0193 |
|  |  | 12 | 0.985 | 0.8843 | 1.0811 | 0.040 | 0.0328 | 0.0485 |
|  | 2011 | 1 | 1.128 | 0.9762 | 1.2943 | 0.037 | 0.0262 | 0.0495 |
|  |  | 2 | 1.426 | 1.2245 | 1.6849 | 0.041 | 0.032 | 0.0501 |
|  |  | 3 | 1.363 | 1.1086 | 1.6189 | 0.050 | 0.0403 | 0.0619 |
|  |  | 5 | 2.123 | 1.7366 | 2.5997 | 0.028 | 0.0192 | 0.0367 |
|  |  | 10 | 3.150 | 2.575 | 3.8615 | 0.002 | -0.00908 | 0.0144 |
|  | Annual (yr 1) | | 1.627 | 1.5384 | 1.7251 | 0.054 | 0.0512 | 0.057 |
|  | Annual (yr 2) | | 1.714 | 1.6339 | 1.7933 | 0.045 | 0.0431 | 0.0478 |
|  | Annual (yr 3) | | 1.483 | 1.4014 | 1.573 | 0.048 | 0.0452 | 0.0507 |
| Xeric | 2008 | 10 | 0.895 | 0.5857 | 1.1989 | 0.083 | 0.0409 | 0.1317 |
|  |  | 11 | 1.294 | 1.0075 | 1.5654 | 0.052 | 0.0382 | 0.0686 |
|  |  | 12 | 1.405 | 1.2486 | 1.5596 | 0.029 | 0.0222 | 0.0371 |
|  | 2009 | 1 | 1.069 | 0.9499 | 1.2034 | 0.069 | 0.0603 | 0.0761 |
|  |  | 2 | 1.027 | 0.8879 | 1.169 | 0.046 | 0.0372 | 0.0557 |
|  |  | 3 | 1.588 | 1.2257 | 1.9973 | 0.045 | 0.0338 | 0.0582 |
|  |  | 4 | 1.303 | 0.9909 | 1.6723 | 0.066 | 0.0532 | 0.0806 |
|  |  | 8 | 1.429 | 0.5044 | 4.1223 | 0.064 | 0.0195 | 0.1062 |
|  |  | 9 | 3.933 | 1.8076 | 6.378 | 0.018 | -0.00323 | 0.0515 |
|  |  | 10 | 1.665 | 1.2971 | 1.9995 | 0.053 | 0.0441 | 0.0643 |
|  |  | 11 | 2.544 | 1.9776 | 3.2752 | 0.015 | -0.00189 | 0.0311 |
|  |  | 12 | 1.030 | 0.9067 | 1.1686 | 0.073 | 0.0599 | 0.0847 |
|  | 2010 | 1 | 0.730 | 0.6593 | 0.8052 | 0.070 | 0.0612 | 0.0791 |
|  |  | 2 | 0.778 | 0.6931 | 0.8721 | 0.071 | 0.0587 | 0.0833 |
|  |  | 3 | 1.023 | 0.8541 | 1.2392 | 0.069 | 0.0565 | 0.0811 |
|  |  | 4 | 1.711 | 1.1916 | 2.434 | 0.046 | 0.0273 | 0.0644 |
|  |  | 5 | 4.959 | 2.9832 | 8.4022 | 0.004 | -0.018 | 0.0271 |
|  |  | 11 | 1.186 | 0.9656 | 1.4416 | 0.055 | 0.0433 | 0.0683 |
|  |  | 12 | 0.828 | 0.7523 | 0.9063 | 0.047 | 0.0412 | 0.0542 |
|  | 2011 | 1 | 0.942 | 0.8362 | 1.0495 | 0.050 | 0.0302 | 0.0664 |
|  |  | 2 | 1.185 | 1.0239 | 1.3785 | 0.045 | 0.0356 | 0.0529 |
|  |  | 3 | 1.332 | 1.0199 | 1.6438 | 0.047 | 0.0355 | 0.0629 |
|  |  | 4 | 3.060 | 2.2183 | 4.1221 | 0.026 | 0.0121 | 0.0399 |
|  |  | 5 | 1.175 | 0.8236 | 1.5534 | 0.047 | 0.0327 | 0.0634 |
|  |  | 6 | 3.379 | 1.7788 | 6.3543 | 0.004 | -0.02 | 0.0277 |
|  |  | 9 | 3.246 | 1.7964 | 5.4571 | 0.019 | -0.00567 | 0.0467 |
|  |  | 10 | 2.234 | 1.5587 | 2.9542 | 0.013 | -0.00221 | 0.0337 |
|  | Annual (yr 1) | | 1.319 | 1.2229 | 1.411 | 0.060 | 0.0565 | 0.0636 |
|  | Annual (yr 2) | | 1.393 | 1.3157 | 1.4621 | 0.052 | 0.0494 | 0.0547 |
|  | Annual (yr 3) | | 1.275 | 1.1958 | 1.3521 | 0.050 | 0.0466 | 0.0527 |
