# Supplementary material for: Cyclic Occurrence of Fire and Its Role in Carbon Dynamics along an Edaphic Moisture Gradient in Longleaf Pine Ecosystems
Source: PLoS One. 2013 Jan 15;8(1):e54045. doi: 10.1371/journal.pone.0054045 (PMC3545999; doi:10.1371/journal.pone.0054045)
Supplement: Table S2 — Distribution of parameters from Reco bootstrap simulations. LCL = lower limit of 90% confidence region, UCL = upper limit of 90% confidence region. (DOCX) [file pone.0054045.s002.docx]

Table S.2. Distribution of parameters from R_eco_ bootstrap simulations. LCL = lower limit of 90% confidence region, UCL = upper limit of 90% confidence region.

|  |  |  | *R_0_* | | | *B* | | |
| --- | --- | --- | --- | --- | --- | --- | --- | --- |
| Site | Year | Month | Estim. Param. | LCL | UCL | Estim. Param. | LCL | UCL |
| Mesic | 2008 | 10 | 1.455 | 0.0898 | 4.7046 | 0.038 | -0.0219 | 0.1851 |
|  |  | 11 | 1.318 | 1.0986 | 1.5655 | 0.048 | 0.0352 | 0.0613 |
|  |  | 12 | 1.138 | 1.0044 | 1.2908 | 0.051 | 0.0425 | 0.0595 |
|  | 2009 | 1 | 1.180 | 1.0628 | 1.3072 | 0.046 | 0.0387 | 0.0532 |
|  |  | 2 | 1.148 | 0.8394 | 1.4637 | 0.050 | 0.0315 | 0.0734 |
|  |  | 3 | 1.315 | 0.9939 | 1.6134 | 0.052 | 0.039 | 0.0681 |
|  |  | 4 | 1.093 | 0.8019 | 1.411 | 0.065 | 0.0495 | 0.0828 |
|  |  | 5 | 2.804 | 1.7013 | 4.098 | 0.029 | 0.01 | 0.0523 |
|  |  | 6 | 3.349 | 1.5979 | 6.9929 | 0.022 | -0.00697 | 0.0514 |
|  |  | 8 | 1.174 | 0.1816 | 6.2642 | 0.077 | 0.00763 | 0.1547 |
|  |  | 9 | 5.713 | 2.6984 | 13.1515 | 0.007 | -0.0295 | 0.0393 |
|  |  | 10 | 1.828 | 1.3851 | 2.2527 | 0.052 | 0.0421 | 0.0659 |
|  |  | 11 | 2.447 | 1.4905 | 3.5475 | 0.032 | 0.00282 | 0.0707 |
|  |  | 12 | 1.587 | 1.2694 | 1.9947 | 0.037 | 0.0171 | 0.0561 |
|  | 2010 | 1 | 0.761 | 0.7055 | 0.8245 | 0.069 | 0.0625 | 0.0761 |
|  |  | 2 | 0.816 | 0.7152 | 0.912 | 0.068 | 0.0578 | 0.081 |
|  |  | 3 | 1.177 | 0.9119 | 1.4985 | 0.056 | 0.0413 | 0.0718 |
|  |  | 4 | 1.605 | 1.0159 | 2.593 | 0.045 | 0.0201 | 0.0688 |
|  |  | 5 | 3.049 | 1.4617 | 5.3043 | 0.026 | 0.00108 | 0.0609 |
|  |  | 11 | 1.162 | 0.6874 | 1.5557 | 0.059 | 0.0391 | 0.0946 |
|  |  | 12 | 0.876 | 0.7597 | 1.0033 | 0.048 | 0.0415 | 0.0552 |
|  | 2011 | 1 | 0.577 | 0.2639 | 0.8894 | 0.115 | 0.0762 | 0.1802 |
|  |  | 2 | 1.158 | 0.9909 | 1.3914 | 0.044 | 0.0321 | 0.0529 |
|  |  | 3 | 1.559 | 1.1204 | 2.0335 | 0.037 | 0.0206 | 0.0575 |
|  |  | 5 | 1.955 | 1.4921 | 2.4843 | 0.031 | 0.02 | 0.0429 |
|  |  | 8 | 2.247 | 0.3574 | 13.3571 | 0.038 | -0.0314 | 0.1085 |
|  |  | 9 | 3.678 | 2.4929 | 5.3538 | 0.001 | -0.0163 | 0.0183 |
|  |  | 10 | 2.293 | 1.6053 | 3.0127 | 0.001 | -0.0172 | 0.0221 |
|  | Annual (yr 1) | | 1.009 | 0.9247 | 1.1034 | 0.075 | 0.0701 | 0.0791 |
|  | Annual (yr 2) | | 1.259 | 1.1898 | 1.3343 | 0.060 | 0.0567 | 0.0622 |
|  | Annual (yr 3) | | 1.269 | 1.1642 | 1.3713 | 0.050 | 0.0461 | 0.054 |
| Intermediate | 2008 | 10 | 3.027 | 2.4654 | 3.7045 | 0.032 | 0.0167 | 0.046 |
|  |  | 11 | 2.910 | 2.4731 | 3.3384 | 0.023 | 0.0129 | 0.035 |
|  |  | 12 | 1.723 | 1.4396 | 2.0875 | 0.019 | 0.00693 | 0.0308 |
|  | 2009 | 1 | 1.132 | 1.014 | 1.2478 | 0.054 | 0.0465 | 0.0616 |
|  |  | 2 | 1.651 | 1.4345 | 1.8873 | 0.023 | 0.0127 | 0.0322 |
|  |  | 3 | 2.297 | 1.8906 | 2.8213 | 0.027 | 0.0163 | 0.0374 |
|  |  | 4 | 1.317 | 0.9585 | 1.6626 | 0.058 | 0.0442 | 0.0758 |
|  |  | 9 | 6.968 | 3.8577 | 12.1759 | 0.000 | -0.023 | 0.0248 |
|  |  | 10 | 2.549 | 2.1673 | 2.9417 | 0.035 | 0.027 | 0.0429 |
|  |  | 12 | 1.456 | 1.2858 | 1.6456 | 0.048 | 0.0385 | 0.0567 |
|  | 2010 | 1 | 1.001 | 0.8107 | 1.1619 | 0.062 | 0.0491 | 0.0812 |
|  |  | 2 | 1.252 | 1.1201 | 1.3708 | 0.056 | 0.0466 | 0.0657 |
|  |  | 3 | 1.016 | 0.8623 | 1.1878 | 0.075 | 0.0648 | 0.087 |
|  |  | 5 | 3.574 | 2.2085 | 5.4983 | 0.020 | 0.00115 | 0.0417 |
|  |  | 8 | 6.601 | 2.4918 | 16.0456 | 0.002 | -0.0332 | 0.0405 |
|  |  | 11 | 2.408 | 1.9086 | 3.0429 | 0.006 | -0.00742 | 0.0193 |
|  |  | 12 | 0.985 | 0.8843 | 1.0811 | 0.040 | 0.0328 | 0.0485 |
|  | 2011 | 1 | 1.128 | 0.9762 | 1.2943 | 0.037 | 0.0262 | 0.0495 |
|  |  | 2 | 1.426 | 1.2245 | 1.6849 | 0.041 | 0.032 | 0.0501 |
|  |  | 3 | 1.363 | 1.1086 | 1.6189 | 0.050 | 0.0403 | 0.0619 |
|  |  | 5 | 2.123 | 1.7366 | 2.5997 | 0.028 | 0.0192 | 0.0367 |
|  |  | 10 | 3.150 | 2.575 | 3.8615 | 0.002 | -0.00908 | 0.0144 |
|  | Annual (yr 1) | | 1.627 | 1.5384 | 1.7251 | 0.054 | 0.0512 | 0.057 |
|  | Annual (yr 2) | | 1.714 | 1.6339 | 1.7933 | 0.045 | 0.0431 | 0.0478 |
|  | Annual (yr 3) | | 1.483 | 1.4014 | 1.573 | 0.048 | 0.0452 | 0.0507 |
| Xeric | 2008 | 10 | 0.895 | 0.5857 | 1.1989 | 0.083 | 0.0409 | 0.1317 |
|  |  | 11 | 1.294 | 1.0075 | 1.5654 | 0.052 | 0.0382 | 0.0686 |
|  |  | 12 | 1.405 | 1.2486 | 1.5596 | 0.029 | 0.0222 | 0.0371 |
|  | 2009 | 1 | 1.069 | 0.9499 | 1.2034 | 0.069 | 0.0603 | 0.0761 |
|  |  | 2 | 1.027 | 0.8879 | 1.169 | 0.046 | 0.0372 | 0.0557 |
|  |  | 3 | 1.588 | 1.2257 | 1.9973 | 0.045 | 0.0338 | 0.0582 |
|  |  | 4 | 1.303 | 0.9909 | 1.6723 | 0.066 | 0.0532 | 0.0806 |
|  |  | 8 | 1.429 | 0.5044 | 4.1223 | 0.064 | 0.0195 | 0.1062 |
|  |  | 9 | 3.933 | 1.8076 | 6.378 | 0.018 | -0.00323 | 0.0515 |
|  |  | 10 | 1.665 | 1.2971 | 1.9995 | 0.053 | 0.0441 | 0.0643 |
|  |  | 11 | 2.544 | 1.9776 | 3.2752 | 0.015 | -0.00189 | 0.0311 |
|  |  | 12 | 1.030 | 0.9067 | 1.1686 | 0.073 | 0.0599 | 0.0847 |
|  | 2010 | 1 | 0.730 | 0.6593 | 0.8052 | 0.070 | 0.0612 | 0.0791 |
|  |  | 2 | 0.778 | 0.6931 | 0.8721 | 0.071 | 0.0587 | 0.0833 |
|  |  | 3 | 1.023 | 0.8541 | 1.2392 | 0.069 | 0.0565 | 0.0811 |
|  |  | 4 | 1.711 | 1.1916 | 2.434 | 0.046 | 0.0273 | 0.0644 |
|  |  | 5 | 4.959 | 2.9832 | 8.4022 | 0.004 | -0.018 | 0.0271 |
|  |  | 11 | 1.186 | 0.9656 | 1.4416 | 0.055 | 0.0433 | 0.0683 |
|  |  | 12 | 0.828 | 0.7523 | 0.9063 | 0.047 | 0.0412 | 0.0542 |
|  | 2011 | 1 | 0.942 | 0.8362 | 1.0495 | 0.050 | 0.0302 | 0.0664 |
|  |  | 2 | 1.185 | 1.0239 | 1.3785 | 0.045 | 0.0356 | 0.0529 |
|  |  | 3 | 1.332 | 1.0199 | 1.6438 | 0.047 | 0.0355 | 0.0629 |
|  |  | 4 | 3.060 | 2.2183 | 4.1221 | 0.026 | 0.0121 | 0.0399 |
|  |  | 5 | 1.175 | 0.8236 | 1.5534 | 0.047 | 0.0327 | 0.0634 |
|  |  | 6 | 3.379 | 1.7788 | 6.3543 | 0.004 | -0.02 | 0.0277 |
|  |  | 9 | 3.246 | 1.7964 | 5.4571 | 0.019 | -0.00567 | 0.0467 |
|  |  | 10 | 2.234 | 1.5587 | 2.9542 | 0.013 | -0.00221 | 0.0337 |
|  | Annual (yr 1) | | 1.319 | 1.2229 | 1.411 | 0.060 | 0.0565 | 0.0636 |
|  | Annual (yr 2) | | 1.393 | 1.3157 | 1.4621 | 0.052 | 0.0494 | 0.0547 |
|  | Annual (yr 3) | | 1.275 | 1.1958 | 1.3521 | 0.050 | 0.0466 | 0.0527 |
